# Multi-omics protein-coding units as massively parallel Bayesian networks: Empirical validation of causality structure

**DOI:** 10.1016/j.isci.2022.104048

**Published:** 2022-03-11

**Authors:** Alberto Zenere, Olof Rundquist, Mika Gustafsson, Claudio Altafini

**Affiliations:** 1 Division of Automatic Control, Department of Electrical Engineering, Linköping University, SE-58183 Linköping, Sweden; 2 Bioinformatics, Department of Physics, Chemistry and Biology, Linköping University, SE-58183 Linköping, Sweden

**Keywords:** Bioinformatics, Mathematical biosciences, Systems biology, Omics

## Abstract

In this article we use high-throughput epigenomics, transcriptomics, and proteomics data to construct fine-graded models of the “protein-coding units” gathering all transcript isoforms and chromatin accessibility peaks associated with more than 4000 genes in humans. Each protein-coding unit has the structure of a directed acyclic graph (DAG) and can be represented as a Bayesian network. The factorization of the joint probability distribution induced by the DAGs imposes a number of conditional independence relationships among the variables forming a protein-coding unit, corresponding to the missing edges in the DAGs. We show that a large fraction of these conditional independencies are indeed verified by the data. Factors driving this verification appear to be the structural and functional annotation of the transcript isoforms, as well as a notion of structural balance (or frustration-free) of the corresponding sample correlation graph, which naturally leads to reduction of correlation (and hence to independence) upon conditioning.

## Highlights

• Protein coding unit: DAG associated with epigenetic and gene information of a protein
• DAGs correspond to Bayesian networks
• Edge absence on a DAG corresponds to conditional independence
• Multi-omics data (ATAC-seq, RNA-seq and mass-spec) are used for DAG validation

## Graphical abstract

**Figure:**
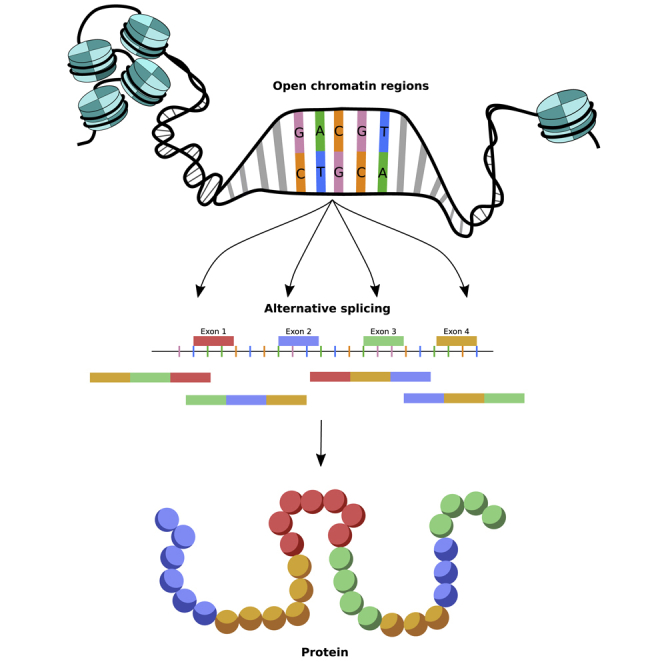

## Introduction

The last 20 years have witnessed an explosion of the number and accuracy of omics techniques, and with them of our possibilities to monitor and understand in a systematic way the events that lead to the expression of genes and proteins (Dihazi et al., 2018; Hawe et al., 2019; Huang et al., 2017; Subramanian et al., 2020). These omics techniques call for integrative models to be developed, and in fact also the number of multi-omics computational solutions proposed in the literature has grown at a stable pace. See the surveys Berger et al. (2013); Bersanelli et al. (2016); Gomez-Cabrero et al. (2014); Meng et al. (2014); and Zeng and Lumley (2018). Most of this literature focuses on system-level methods in which genome-wide models are developed and investigated. Typical examples are gene regulatory networks (Duren et al., 2017; Greenfield et al., 2013; Huynh-Thu and Sanguinetti, 2019; Miraldi et al., 2019; Siahpirani and Roy, 2017), protein-protein interaction networks (DeLas Rivas and Fontanillo, 2010; Vogel and Marcotte, 2012), or combinations of these layers with genetic interactions, methylation, genome-wide association studies, signaling, and so on (Buetti-Dinh et al., 2020; Fu et al., 2017; Godsey, 2013).

The approach we take in this article is not aimed at developing novel system-level models, but rather at integrating various omics into fine-graded models of transcription and translation at the level of single gene/protein. The omics we consider are epigenomics (ATAC-seq), transcriptomics (RNA-seq), and proteomics (mass spectrometry), all performed on primary human naive CD4^+^T-cells during early T-helper type 1 (T_H_1) cell differentiation. ATAC-seq (Assay for Transposase-Accessible Chromatin sequencing) is one of the most popular high throughput techniques for chromatin accessibility profiling (Yan et al., 2020). The regions (called peaks) of open chromatin identified by the method can be associated with the nearest gene and play an important role in its regulation, as the binding of transcription factors to DNA is enabled (or facilitated) by chromatin accessibility. RNA-sequencing at sufficient depth ($4\;\cdot \;10^{7}$ reads per sample in our case) allows us to quantify gene expression at the level of alternative splice variants of a gene. For this reason, we have decided to consider in our model the expression of these splice variants in place of a single lump value describing gene expression. Annotated ATAC-seq peaks can be assigned with reasonable accuracy also to the splice variants of a gene, by labeling them according to their transcription start site. To measure protein abundance we used mass spectrometry. As this technique is not sensitive enough to detect protein isoforms in a systematic way (Blencowe, 2017; Tress et al., 2017), in our model all splice variants of a gene are necessarily associated with the protein that the corresponding gene is coding for.

While these types of data have been around for a while, understanding how to combine them in a constructive way is still problematic because of the low RNA-protein and RNA-ATAC correlations (Duren et al., 2017; Fortelny et al., 2017; Vogel and Marcotte, 2012), and because of the limited knowledge about how to deal with splice-level transcripts in a systematic way (Floor and Doudna, 2016). The aim of this article is to contribute to these topics.

**Figure 1:**
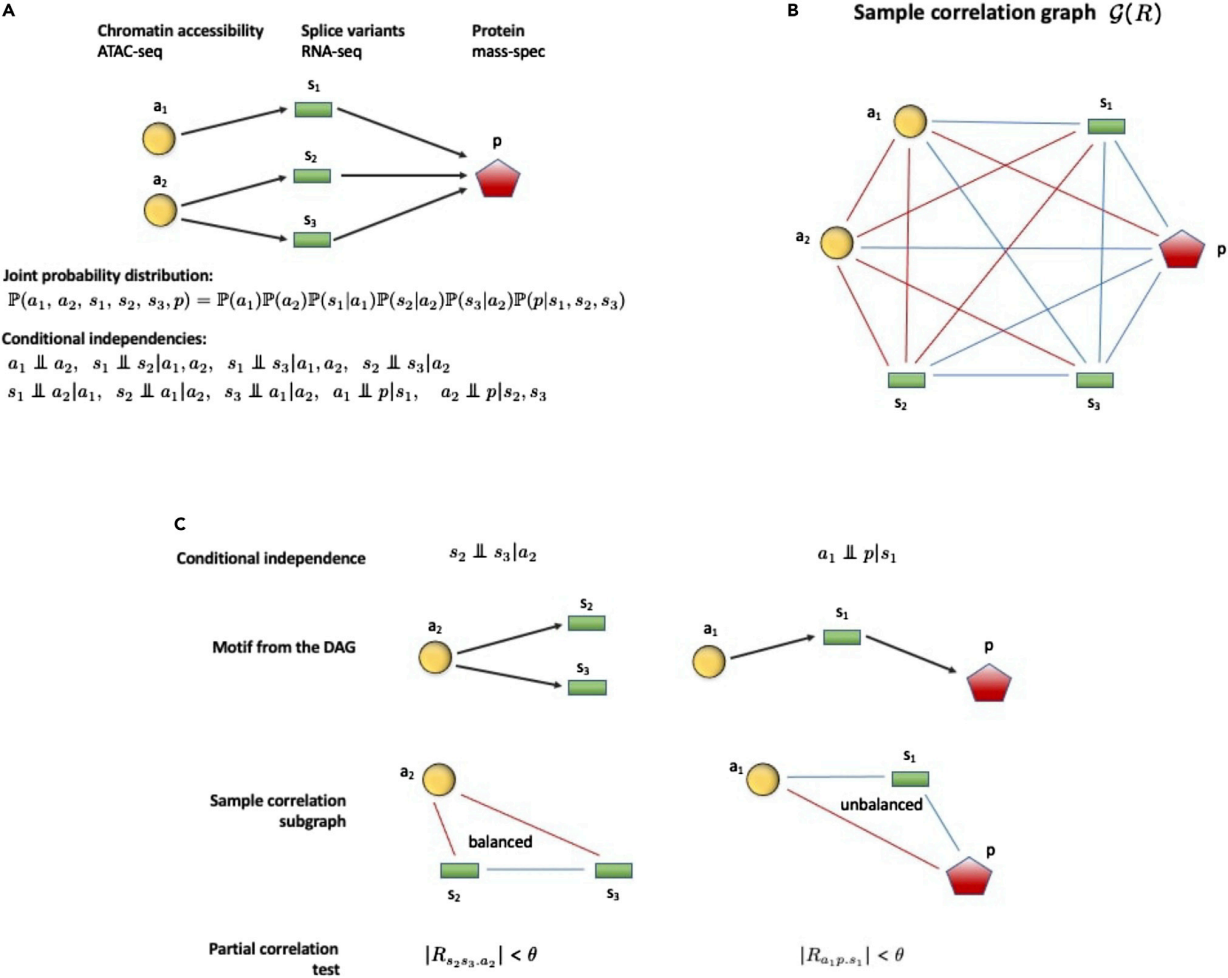
Introduction. Conceptual workflow of the article (A) The causal dependencies considered in a protein-coding unit can be represented as a DAG. A Bayesian network can be associated with the DAG, with joint probability distribution that factorizes according to the DAG. A number of conditional independence relationships follow (notice that some of the conditioning sets can be reduced). (B) The sample correlation graph G(R) corresponding to the DAG. Blue (resp. red) edges represent positive (resp. negative) entries of *R*. In this case G(R) is not balanced. (C) Checking conditional independence on the data: examples. The variables involved in a conditional independence form a motif on the DAG, to which is associated a subgraph of G(R). If this subgraph is balanced then typically the correlation contracts upon conditioning, often leading to an empirical validation of the predicted conditional independence.

Combining the three layers of data with bioinformatic analysis, we construct a map from chromatin accessibility to splice variants and then one from splice variants to protein. We call such a combined map a *protein-coding unit*. Since causality flows unidirectionally from chromatin accessibility to transcripts and then to proteins, the map can be represented as a directed acyclic graph (DAG), see Figure 1A. As the protein-coding units associated with different genes/proteins are essentially disjoint when only the aforementioned variables are considered, the entire genome can be modeled as a large set of independent DAGs, which can be analyzed in parallel. For this choice of model we can work in an extremely advantageous regime: 1) The interaction graph (i.e., the DAG) can be assumed to be known; 2) all variables of interest (i.e., the nodes of the DAG) are experimentally measured.

A class of models that is particularly suitable to deal with DAGs is Bayesian networks (Friedman, 2004; Huynh-Thu and Sanguinetti, 2019; Kalisch and Bühlmann, 2007; Koller and Friedman, 2009; Lauritzen, 1996; Maathuis et al., 2010; Pearl, 1988). A Bayesian network is actually defined as a DAG with a random variable assigned to each node. When the topology of the graph is known, then the joint probability distribution of the Bayesian network factorizes according to the DAG. Full measurement of the variables means that we have the opportunity to verify if indeed the sample distribution respects the structure predicted by the DAG. In fact, in a Bayesian network, absence of an edge between two nodes can be expressed as a conditional independence relationship among the variables (Koller and Friedman, 2009; Lauritzen, 1996). Hence, checking the DAG factorization is equivalent to checking how many of the corresponding conditional independencies are verified by the data, test which can be carried out by computing partial correlations. We show in the article that the data indeed verify many of the predicted conditional independencies, meaning that the predicted structure of the protein-coding units is largely validated by our data. The verification of conditional independencies is particularly high for protein-coding units directly involved in the cellular process that our data are describing, T-cell differentiation, and even more for T_H_1 cell differentiation.

Among the factors leading to verification of conditional independence, the functional and structural annotation of splice variants of a gene appear to play a major role, thereby confirming the idea that “principal” transcript isoforms are indeed an important factor behind protein synthesis, see Gonzalez-Porta et al. (2013); Ezkurdia et al. (2015). The relevance of such isoforms is here cross-validated by using conditional independence relationships to determine a dominant splice variant in each protein-coding unit. The method we propose provides a ranking of the splice variants of a gene even when standard criteria, like RNA abundance, fail to do so.

Apart from these results, a novel contribution of this article consists in identifying a property of sample data that most likely leads to a validation of a conditional independence relationship. To our DAGs we can associate a matrix of sample correlations *R*, and the corresponding signed sample correlation graph G(R). We show in the article that when the subgraph of G(R) associated with a conditional independence is structurally balanced (i.e., all its cycles have an even number of negative edges), then it is much more likely that the corresponding conditional independence is validated by the sample data. Structural balance (hereafter referred to simply as “balance”) is a well-known concept in graph theory (Harary et al., 1965). It is related to the notion of frustration-free graph in Ising spin glasses (Facchetti et al., 2011; Mezard and Montanari, 2009; Mezard et al., 1986), and to the idea of coherence in biological networks (Alon, 2007). Its interpretation in the present context is similar to that of a “parity check” in error correcting codes (Cover and Thomas, 2006; Gallager, 1963; Mezard and Montanari, 2009): it provides an internal check of consistency among the sample data involved in the graph. What we show in the article is that balance most often leads to contraction of the correlation upon conditioning, i.e., to a partial correlation that shrinks toward 0, and hence toward “exact” conditional independence.

The observation that balance corresponds to consistent sample data is in our knowledge novel for the field of Bayesian networks. Somewhat related concepts of “internal consistency” have been used for graphical models in Lauritzen et al. (2019); Slawski and Hein (2015) in relationship to a property which we call inverse balance, but which is called signed multivariable total positivity of order 2 (signed MTP_2_) in Lauritzen et al. (2019), and deals with the concentration matrix associated with *R*. The latter property is more restrictive than balance, however it can be shown that for it the aforementioned idea of contraction upon conditioning holds systematically. The impact of both balance and inverse balance on the empirical validation of the structure of the protein-coding units is analyzed in detail in the article.

## Results

The 4797 protein-coding units obtained from our data are represented as DAGs and treated as a parallel set of Bayesian networks, as described in the Methods and in Figure 1. Our aim is to check consistency between the factorization of the joint probability distribution predicted by the structure of the DAGs and our sample data for ATAC-seq (variable *a* in our notation), splice-level RNA-seq (variable *s*) and mass-spec protein abundance (variable *p*), i.e., to check to what extent also the empirical sample distributions factorize according to the DAGs, and what are the implications for our protein-coding units. For that, it is convenient to express the factorized probability distribution of a DAG as a set of conditional independence relationships (also called Markov conditions) among the nodes of the DAG, as described in the Methods and summarized in Table 1. See also Figure 1 for an example.

**Table 1:** Total number of predicted and verified conditional independencies

Markov c.i	Total	Type			
		a--a	s--s	a → s	a → p
Total	151,362	3,741	122,094	16,607	8,920
Verified	66,264	2,167	41,292	16,192	6,613
Non-verified	85,098	1,574	80,802	415	2,307

These conditional independencies can be classified according to the type of edge involved. More than 90% of the non-verified conditional independencies are of type s - s, while for the a → s and a → p the verification rate is much higher

### Conditional independence verification via partial correlations

**Figure 2:**
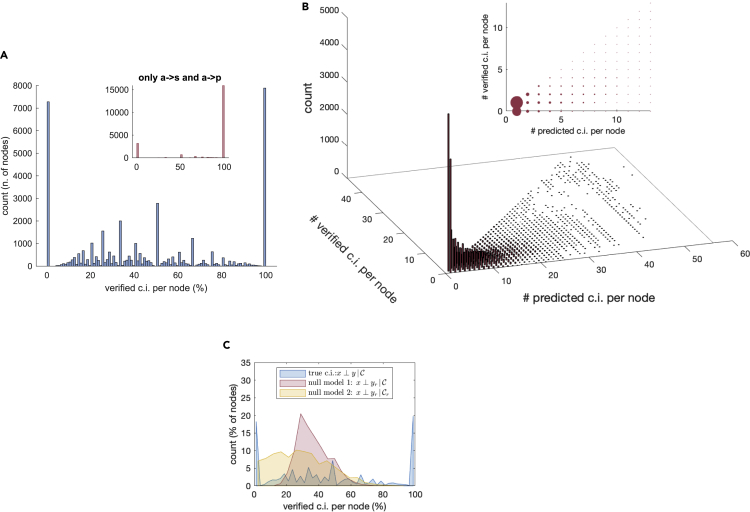
Conditional independence verification via partial correlations. Verifying conditional independencies (A) Ratio between the number of verified and predicted conditional independencies for each node (*a*, *s*, or *p*) of a DAG. Inset: same ratio but when considering only the conditional independencies between different layers, a→s and a→p. (B) The histogram reports the n. of verified and predicted conditional independencies, this time binned according to the n. of predicted conditional independencies (inset: alternative view of the same quantities, with size of the dots proportional to the height of the bars). (C) Distribution of successful conditional independences in the true model (i.e., x⊥y|C, blue), in a null model in which *y* is replaced by a randomly chosen $y_{r}$ (red), and in a null model in which both *y* and C are replaced by randomly chosen $y_{r}$ and $C_{r}$ (of the same cardinality of C, yellow).

With the data we have available, a correlation-based verification test can be carried out exhaustively on the entire set of $1.5\;\cdot \;10^{5}$ predicted conditional independencies, using sample partial correlations. In formulas, a conditional independence between variables *x* and *y* with conditioning set C is expressed as x⊥y|C, where *x* and *y* are any of *a*, *s*, and *p*, localized on the same DAG. Verification of the conditional independence corresponds to the partial correlation $R_{xy.C}$ being small enough: $|R_{xy.C}|\;<\;\theta$, where θ is a threshold provided by a Fisher z-test, see STAR Methods for more details. As can be seen in Figure 2A, for a significant fraction of nodes the conditional independencies are indeed verified by the sample partial correlation test. In particular, the number of nodes with 100% and with 0% of successes is resp. 19.5% and 17.9%, while 44.3% of the nodes have at least 50% of confirmed conditional independencies. The verification is particularly successful for conditional independencies between different “layers” of the DAG, which are by far the most important types of independencies when it comes to understanding the structure of the DAGs, see the Discussion section for more details. For them the overall percentage of successes is 89.3%, see Table 1 and inset in Figure 2A. In practice, this result means that the vast majority of causal independencies among the *a*, *s* and *p* variables predicted by the structure of the Bayesian networks are indeed confirmed by the data.

**Figure 3:**
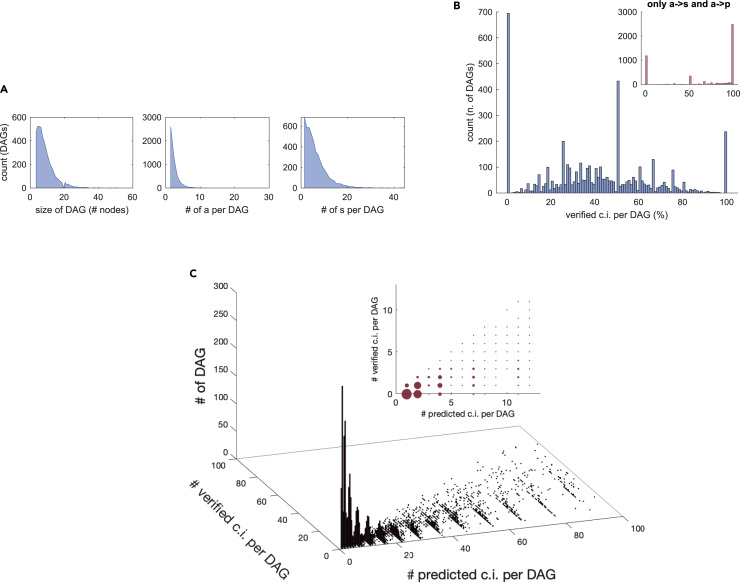
Conditional independence verification via partial correlations. Verifying conditional independencies on DAGs (A) Left: size the DAGs; middle: number of ATAC peaks per DAG; right: number of splice variants per DAG. (B) Ratio between number of verified and number of predicted conditional independencies per DAG. Inset: same ratio but when considering only the conditional independencies between different layers, a→s and a→p. (C) Histogram of the n. of verified and predicted Markov conditions, binned according to the n. of predicted conditional independencies on a DAG (inset: zoomed alternative view of the same quantities, with size of the dots proportional to the height of the bars).

It is also possible to aggregate the statistics of the Markov conditions according to the DAGs to which a node belongs. The size of the DAGs varies as shown in Figure 3A, and the total number of predicted and verified conditional independencies is shown in Figure 3C. The maximum number of predicted (resp. verified) conditional independencies on a DAG is 1201 (resp. 915). For 14.5% of the DAGs no conditional independence is verified, while for 4.9% all are verified. When focusing only on the a→s and a→p classes of conditional independencies the corresponding percentages are 24.7% and 51.4%, see inset in Figure 3B.

### Validation based on null models

To evaluate whether the level of confirmation of the causal dependencies we obtain from the data is indeed meaningful for our protein-coding units, we compared the true distribution of the verified conditional independencies with two null models. The first null model is obtained by replacing *y* in an expression like x⊥y|C with a randomly chosen $y_{r}$ (not necessarily located on the same DAG). In the second null model, both *y* and C are instead replaced by randomly chosen $y_{r}$ and $C_{r}$ (of the same cardinality as C and also in this case not necessarily on the same DAG). The latter null model expresses the probability of obtaining a conditional independence by pure chance, while the former null model describes how much of the information on *x* is captured by C (recall that C is interpreted either as a set of parents of *x* for local Markov conditions or as a d-separating set for global Markov conditions), see STAR Methods. As can be seen in Figure 2C, the first null model ($x\;⊥\;y_{r}|C$) shifts the histogram to the right with respect to the second ($x\;⊥\;y_{r}|C_{r}$). Although conditional independence is detectable in average only in 36.8% of the trials against 29%, the difference is significant (2-sample t-test, pvalue ∼0). The distribution for the true conditional independences is essentially bimodal, with an average of successes of 46.4%. Also this is a statistically significant difference against the 36.8% of the best null model.

### Validation based on the ontology of the protein-coding units

As our experiments describe a CD4^+^T-cell differentiation process toward T_H_1 cells, we reasoned that the signal-to-noise ratio in our data should be particularly high for the protein-coding units directly involved in such process. To verify this hypothesis, we compiled a list of genes involved in T-cell differentiation at large, and a second list more specific to T_H_1 differentiation (see STAR Methods) and checked to what extent the conditional independencies described above are verified in these subsets of protein-coding units. Quite remarkably, we observe that nearly all statistics indeed improve, as can be seen in Table 2. In particular, the verification of the a→p conditional independencies (the most significant class of conditional independencies, given the structure of our DAGs) passes from 74.1% of the whole set of DAGs to 83% for the T-cell related DAGs, to 89.4% for T_H_1 related DAGs. All these improvements are statistically significant (Fisher exact test, see Table 2 for the pvalues). Analogous considerations hold also for other classes of conditional independencies (a−a and s−s), see Table 2. As an alternative analysis, we also checked the ontological enrichment of the two subsets of protein-coding units representing 0 and 100% of verified conditional independencies of type a→s and a→p shown in the inset of Figure 3B. Also, in this case, the second subset is enriched in pathways related to T-cells and their differentiation, while the first is not, see Figure S1 in the Supplementary Information.

**Table 2:** Fraction of verified conditional independencies according to type (see Table 1) and according to protein ontology

Fraction of verified c.i	n. DAGs	Type			
		a--a	s--s	a → s	a → p
All DAGs	4797	57.9%	33.8%	97.5%	74.1%
T cell related DAGs	148	65% (0.01)	40% (10^−13^)	97.1%	83% (10^−5^)
T_H_1 related DAGs	34	66.7%	39.7%(6 · 10^−5^)	97.3%	90.2% (3 · 10^−5^)

P-values from an exact Fisher test relative to the “all DAGs” classification are reported when the result is at a significance level of at least 0.05.

### Determining the dominant splice variant behind protein synthesis

**Figure 4:**
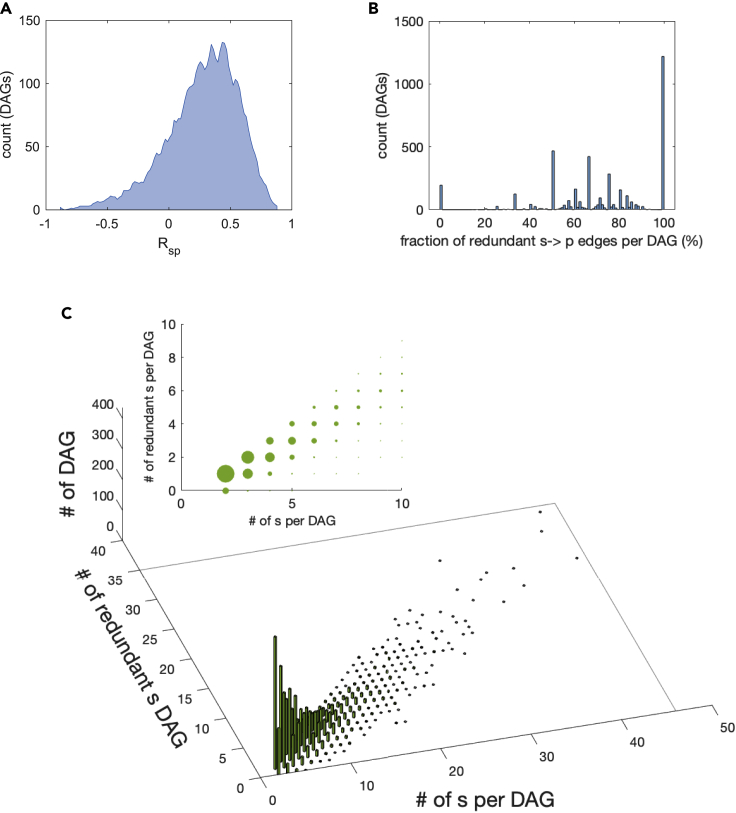
Determining the dominant splice variant behind protein synthesis. Finding the dominant splice variant on DAGs (A) histogram of the correlation between splice variants and corresponding protein (only the best correlating splice variant is considered for each protein). (B) Fraction of alternative splice variants in a DAG which can be explained by a single “dominant” splice variant $s_{i}$, i.e., fraction of $s_{j}$ such that $s_{j}\;⊥\;p|s_{i}$. 100% means that n−1 of the *n* splice variants can be explained by a single splice variant. (C) Histogram of the number of explained splice variants vs number of splices in a DAG, for all DAGs (inset: the same quantity is shown, with width of the dots proportional to height of the bars).

Another analysis that can be performed on our data using conditional independence relationships consists in computing the splice variant that can be considered the most important (we call it “dominant”) in order to “explain” the profile of a protein, and how many splice variants can instead be considered “redundant”, in the sense that their contribution can be explained by the presence of a dominant transcript isoform for that protein. For this, we follow the approach already used in the literature for reconstructing gene regulatory networks (de la Fuente et al., 2004; Schäfer and Strimmer, 2005; Soranzo et al., 2007; Zampieri et al., 2008), namely, on each DAG we search for a splice variant $s_{i}$ such that for all other $s_{j}$ on the same DAG it is $s_{j}\;⊥\;p|s_{i}$, i.e., $|R_{s_{j}p.s_{i}}|<\theta$. Of the 4111 DAGs having more than one splice variant, 29.6% have a single dominant splice isoform, and in 88.4% of the cases a single splice can explain 50% or more of the existing alternative splice variants for that protein, see Figure 4. Also, this test is significant against randomizations. Notice that when restricting to T_H_1-related genes, the percentage of protein-coding units having a single dominant splice isoform grows to 36.4%.

### Factors behind conditional independence: splice function and TF

**Figure 5:**
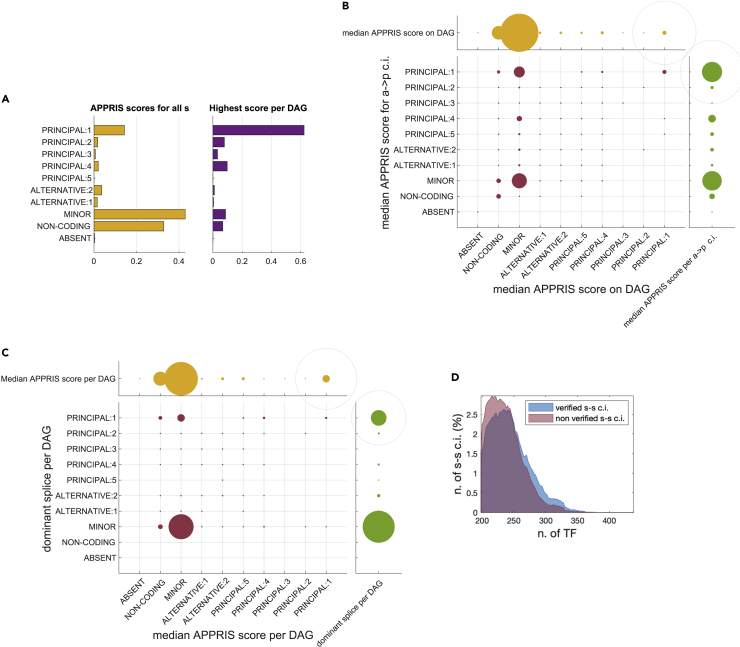
Factors behind conditional independence: splice function and TF. Role of splice annotation and TF (A) To each splice isoform is associated a score obtained from the APPRIS database. The distribution of all scores for the ∼27K splices is shown in the yellow bars. For around 62% of all DAGs a splice labeled PRINCIPAL:1 is present (violet bars). (B) Checking APPRIS scores on the a→p conditional independencies. The median APPRIS score of the *s* that lead to a a→p conditional independence (green) is compare to the median APPRIS score of all the *s* on the DAG (yellow). Dot size is proportional to fraction (gray circle is 100%). As can be seen on the red dots, the median APPRIS score of the observed *s* is systematically higher than the median APPRIS score of the corresponding DAG, meaning that in general *s* with high APPRIS scores (and, in particular, PRINCIPAL:1 splice variants) are more reliable predictors of a conditional independence a→p. (C) Dominant splice variant and APPRIS score. Green disks: APPRIS classification of the dominant splice for each DAG. Red disks: distribution of the dominant splice (according to conditional independence) based on the median APPRIS score for the splices of the corresponding DAG. Yellow disks: median APPRIS score per DAG. (D) Comparing the number of putative TF binding sites on the splice variants involved in verified and non-verified conditional independencies of s−s type.

It is often affirmed, see e.g., Djebali et al. (2012); Ezkurdia et al. (2015); Gonzalez-Porta et al. (2013) (although not without controversy, see Blencowe (2017)), that many transcripts classified as splice variants of a gene are not translated, and that many more contribute to protein synthesis only with a minor probability. To this end, the database APPRIS (Rodriguez et al., 2018) provides a classification of all transcript isoforms of a gene based on annotation and conservation. One may then wonder to what extent the successfully verified conditional independencies we obtain reflect the importance of the functional annotation of the corresponding splice variant. A test can be carried out for some of the classes of conditional independencies listed in Table 1, using the APPRIS ranking of splice variants described in the Methods. For instance, for the a→p conditional independencies, from Figure 5B, it can be seen that of the 6613 validated conditional independencies 2018 (30.5%) correspond to a median APPRIS score which is the highest possible, i.e., the observed *s* for the conditional independence a⊥p|s are labeled PRINCIPAL:1 in APPRIS. The corresponding percentage for an equal number of randomly chosen *s* on the same DAG is 11%, meaning that the result is statistically significant. In other words, the transcript isoform that tends to explain most of the correlation between *a* and *p* happens often to be the splice variant that is labeled principal for that gene in APPRIS.

A similar test cannot be carried out for the a→s and s−s classes of conditional independencies, as for them the transcripts do not appear in the conditioning set C. It can be done, however, when we seek for the dominant splice of a protein-coding unit, as conditioning in this case is over *s* (i.e., over $s_{i}$ in the expression $s_{j}\;⊥\;p|s_{i}$). When we compute the dominant splice variant according to the method described above, we see that in around 28% of the DAGs (42% if we restrict to the DAGs admitting a splice variant classified as PRINCIPAL:1) indeed the dominant splice has an associate score equal to PRINCIPAL:1 (green dots in Figure 5C), i.e., the splice able to alone explain the most of the profile of the protein corresponds to the most important splice according to the current structural and functional knowledge of the proteins. This result is highly significant (Fisher exact test, pvalue of $5\;\cdot \;10^{-13}$).

As an alternative test for the s−s class of conditional independencies (which alone gives more than 90% of the non-verified conditional independencies), it is possible to compute a set of Transcription Factors (TF) potentially associated with each transcript according to a footprinting analysis, as described in the STAR Methods. We can then observe that there is a noticeable difference between splice variants involved in s−s conditional independencies that are verified by the data and those that are not. Namely the former tend to have a higher number of putative TF than the latter, see Figure 5D. This difference is significant (2-sample t-test, p-val ∼0).

### Coupling the protein coding units: shared peaks and protein complexes

Genes and proteins take part in regulatory networks characterized by a multitude of interactions, some of which involve multiple protein-coding units. For instance, protein-coding units may be coupled by the presence of shared chromatin peaks, intended as peaks that influence splices located on different protein coding units (i.e., splices associated with different genes, and hence to different proteins), see Figure S4A. In our data 876 (i.e. ∼3%) peaks belong to this category. To analyze the effects brought by a shared peak, we can consider the joint DAG containing the peaks, splices and proteins of the two different protein units, see the example in Figure S4A. This form of coupling leads to additional conditional independencies in the joint DAG. For example, in the case of the DAG in Figure S4A, we can consider the conditional independencies $s_{12}\;⊥\;s_{21}|a_{2}$, and $p_{1}\;⊥\;p_{2}|\left\{s_{11},s_{12}\right\},$ which do not exist when we consider the two protein-coding units separately. In particular, it is interesting to note that considering interactions between protein-coding units introduces a new class of conditional independencies, that of p−p.

We have systematically tested the set of conditional independencies that arise from the presence of shared peaks, which can be of two types:

$$s_{1i}\;⊥\;s_{2j}|a_{comm},\;\;p_{1}\;⊥\;p_{2}|S_{1},$$

where $s_{ki}$ indicates the *i-th* splice that belongs to the protein unit of $p_{k}$; $a_{comm}$ represent the peaks in common between $s_{1i}$ and $s_{2j}$; and $S_{1}=\left\{s_{11},s_{12},\ldots \right\}$ is the set of splices associated with $p_{1}$ (the situation is specular if we take the set of splices associated with $p_{2}$).

Our analysis shows that ∼29% of these new conditional independencies in the joint DAG are verified by the data, see Table S1, which is a similar value to what was observed for the set of “single-DAG” s−s conditional independencies in Table 1. On the other hand, it is interesting to note that only around 20% of the conditional independencies between proteins is validated by the data. We can interpret this as a sign that the causal links introduced by the shared ATAC peaks do not propagate in a significant way to protein co-regulation.

Another potential form of interactions among our protein-coding units is due to the existence of protein complexes. These introduce another type of interaction in the model, in the form of an undirected p−p edge, see Figure S4B. In terms of probabilistic graphical models, the resulting graph is no longer a Bayesian network but rather a mixed directed/undirected (acyclic) graph (Koller and Friedman, 2009), but for our purposes we can treat it in an analogous way.

To analyze this form of interaction, we have downloaded the list of protein complexes from the database CORUM (Giurgiu et al., 2019), which contains 4274 manually annotated protein complexes from mammalian organisms. Out of these, 1175 complexes correspond to at least a pair of proteins in our data. What we observe is a strong dependence at protein level. For example, proteins that belong to the same complex display a significantly higher correlation compared to random protein pairs, see Figure S4C. This is not unexpected, as subunits of a protein complex should be expressed in fixed stoichiometric ratios.

Moreover, proteins that belong to the same complex rarely satisfy the conditional independence of type $p_{1}\;⊥\;p_{2}|S_{1}$: in only 14% of pairs the condition is satisfied (compared to 20% for random pairs). Even more, when we select only proteins that belong to a protein complex and also share a common peak, only two out of 28 pairs are conditionally independent.

However, the same dependence observed for subunits of a protein complex does not seem to exist at splice level, where we observe correlation values similar to random pairs of splices, see Figure S4C. This observation suggests that the abundance of proteins in the same complex may not always be regulated at the level of transcription, but could be rather due to post-translational modifications, as already described in Eisenberg et al. (2018); Ross et al. (2021).

### Balance as a proxy for conditional independence

It is intriguing to try to understand if there is also any property of Bayesian networks that can be exploited to predict success or failure in our empirical verification of conditional independences. In order to do so, we need to introduce the concept of balance of the signed graph G(R) induced by the sample correlation *R* on a set of nodes, see STAR Methods.

**Figure 6:**
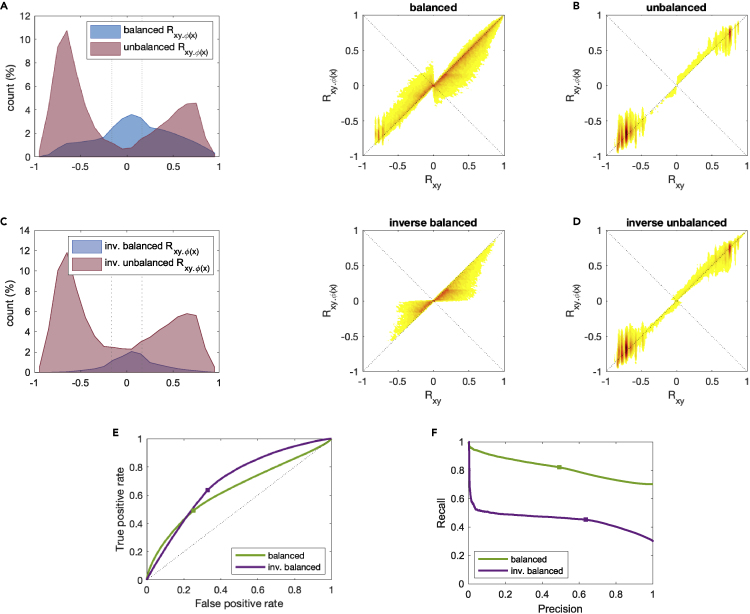
Balance as a proxy for conditional independence. Balance and inverse balance (A) Distribution of the sample partial correlations $R_{xy.C}$, classified according to the balance or unbalance of the corresponding sample correlation graph. The vertical dotted lines correspond to ±θ (significance threshold). Balanced $R_{xy.C}$ tend to be concentrated around 0, while unbalanced $R_{xy.C}$ tend to stay away from 0. (B) Sample density heatmap of the pair $\left(R_{xy},\;R_{xy.C}\right)$ for the balanced (left) and unbalanced (right) cases. In the balanced cases, normally it is $\left|R_{xy.C}|<|R_{xy}\right|$, while no such rule holds in the unbalanced cases. (C) Same histogram as in (A), but now classified according to inverse balance. The pattern is similar to (A), but now the fraction of inverse balanced cases is lower and that of the inverse unbalanced cases higher. (D) Same density plot as in (B), but for inverse balance. The inequality $\left|R_{xy.C}|<|R_{xy}\right|$ is now obeyed systematically in the inverse balanced cases, i.e., conditioning always leads to contraction. (E) ROC curves for balance and inverse balance, as the threshold *θ* is varied. The square marker corresponds to the threshold for θ chosen in the article. AUC for balance is 0.64, while for inverse balance it is 0.69. (F) Precision-recall curves for balance and inverse balance, as the threshold *θ* is varied. AUC for balance is 0.81, while for inverse balance it is 0.45.

If we classify the $∼4\cdot 10^{5}$ partial correlations required for checking all Markov conditions into balanced and unbalanced according to the corresponding correlation graph, then we see a striking difference: the partial correlations $R_{xy.C}$ taken from unbalanced graphs tend systematically to stay away from 0, a pattern not visible in those taken from balanced correlation graphs, see Figure 6A. Consequently, unbalanced graphs seldom lead to verification of conditional independencies (i.e., to $|R_{xy.C}|<\theta$), and in fact in the vast majority of cases (82.5%), verification of a Markov condition is associated with balance of the correlation graph formed by the corresponding variables, see Table 3.

**Table 3:** Classification of conditional independencies into balanced and inverse balanced

Markov c.i	Total	Balance		Inverse balance	
		balanced	unbalanced	balanced	unbalanced
Total	151,362	106,022	45,340	47,055	104,307
Verified	66,264	54,657	11,607	30,868	35,396
Non-verified	85,098	51,365	33,733	16,187	68,911

Both balance and inverse balance help in verifying conditional independencies. Balance is broader and less selective (many more “true positives” than “false negatives”, but also many “false positives”. Inverse balance is more selective, but has lower inference power (less “false positives” and many “true negatives” but also less “true positives”). Overall, inverse balance is slightly more accurate: 7% less “errors” (“false positives + false negatives”) than balance.

### Inverse balance as a more stringent proxy for conditional independence

If instead of the sample correlation matrix *R* we consider the corresponding sample concentration matrix $J=R^{-1}$, then an analogous notion of balance can be set up also for G(J). We call this inverse balance, see STAR Methods for details. If we compare the statistics of verified conditional independencies for balance and inverse balance (Table 3), then we see that while balance is able to capture a larger fraction of verifications, and in fact the number of confirmed conditional independencies associated with unbalanced G(R) is low (“false negatives”, 17.5%), it is also producing a large number of “false positives” i.e., balanced motifs that do not lead to verifications of a conditional independence. On the contrary, inverse balance can verify around half of the conditional independencies confirmed by balance, but the number of concentration graphs that are inverse balanced and that are not verified is around a third of the balanced cases, see Table 3. Treating balanced + non-verified and unbalanced + verified as “errors”, then inverse balance produces around 7% less errors than balance. This is confirmed by the ROC-like curves we obtain if instead of keeping a fixed threshold θ for acceptance of a verification we vary it continuously (a more thorough explanation is in the STAR Methods), see Figure 6E. AUC for balance is 0.64, while for inverse balance it is 0.69. However, if we look at the Precision-Recall curve built in an analogous way, Figure 6F, we see that now the AUC for balance is 0.81, while that for inverse balance is only 0.45.

In short, balance is less restrictive but also less precise (it has lower positive predictive value but higher negative predictive value) than inverse balance in verifying the conditional independencies predicted by the DAGs structures associated with the protein-coding units. Both concepts are however extremely useful in screening large sets of dependencies, and in checking the presence of edges in the corresponding Bayesian networks.

## Discussion

The multiple high-throughput omics data we have available for this study allow us to characterize with reasonable precision several of the crucial steps of the complex process that leads to the synthesis of proteins in higher organisms: ATAC-seq data allow to estimate the chromatin accessibility state in the area of the DNA surrounding the gene of interest, RNA-seq data provide us a quantification of gene expression at the level of alternative splice variants, and mass spectrometry based proteomics can measure the abundance of the corresponding gene products. Combining together these measurements means looking at a fine-graded description of the transcriptional/translational events that lead to the synthesis of a protein. A key contribution of this article is to show that the combination of these multi-omics data can give rise also to novel high-resolution mathematical models.

Importantly, the physical interactions used in these models have a natural DAG structure, suited to be modeled using tools such as Bayesian networks. Even more importantly, the resulting Bayesian networks have a manageable size, and the regime in which they are placed is not the highly unconstrained one (more degrees of freedom than data) normally needed in gene regulatory network inference (Schäfer and Strimmer, 2005; Soranzo et al., 2007), but its opposite. In fact, in our case, each variable is measured and we do not need to learn the graph, as the topology of the Bayesian network itself is available through gene sequence annotation: the ATAC-seq peaks can be assigned with reasonable accuracy to specific splice variants of a gene based on the transcript start site of each splice, and the alternative splicings of a gene are also well characterized. The translation from alternative splices to proteins is perhaps the step that carries the most uncertainty, the limiting factor being the low precision and the limited coverage of current mass spectrometry proteomics. In particular, the limited sensitivity of this technique does not allow in general to identify protein isoforms (Blencowe, 2017; Tress et al., 2017), hence making it impossible to associate the protein signals we have with the specific transcript isoforms.

It is therefore quite remarkable that with no other information than conditional independence relationships among the alternative splice variants of a gene, in around 25–30% of the cases we can determine the splice variant which is classified as principal according to the current annotation of structure, functions and conservation of each transcript isoform. In other words, principal splice variants tend to explain a considerable fraction of the expression of the alternative splices, even though this explanation is not related to abundance, and it is not directly visible in the splice-splice correlation, nor in the splice-protein correlation, but rather at the partial correlation level (i.e., in the conditional dependencies among the variables). In fact, when we classify the correlations between *s* and *p* according to the APPRIS scores, then no particular trend emerges. In Figure S2B, the histogram for PRINCIPAL:1 splice variants is not distinguishable from the others. The splice variants classified as dominant according to our conditional information approach have some overlap with the most abundant splice in our sample, see Figure S2A. Since correlations and partial correlations are invariant to amplitude rescaling, this provides (at least to some extent) a cross-validation of the idea that the most abundant splice isoform is also the most relevant one for translation (Ezkurdia et al., 2015). This appears to be especially true for the protein-coding units containing a low number of splice variants, see Figure S2C. In 23.8% of the DAGs, the dominant splice isoform corresponds also to the splice having the highest correlation with the corresponding protein, see Figure S2B. However, a straightforward relationship (like the one provided by RNA-seq abundance) between a single splice isoform and the corresponding protein appears to be missing for the remaining 70–80% of the proteins we have analyzed. For them, no single feature of the transcript and of its annotation appears to be enough to determine which splice variant is playing the most important role in protein synthesis. Here our approach may provide a novel way to infer the importance of splicing events on translation and protein synthesis.

When we zoom in on the protein-coding units directly involved in T_H_1 differentiation, then, apart from proteins having a trivial DAG (e.g., *IFNG* [Interferon-γ, the main product of T_H_1 cells] and *IL27RA* [Interleukin-27 receptor α chain, involved in T_H_1 differentiation], both having a single transcript isoform, which is of course also principal), we can find several cases of interest. For instance, of the 11 splice isoforms of *STAT4* (a key regulator of T-helper cell differentiation), only four are retained in the DAG after filtering out transcripts that lack ATAC peaks and/or those labeled as non-coding in APPRIS. All of these four transcripts have similar expression, but only two are labeled as PRINCIPAL:1 in APPRIS. One of the two corresponds to the dominant splice we identify through conditional independence analysis. In several examples the various classification criteria that we are discussing (RNA-seq abundance, APPRIS score and our test for dominance) are all consistent. Consider for instance the gene *TBX21*, a Tbox transcription factor thought to be the main driver of T_H_1 differentiation. Of the two transcript isoforms of *TBX21*, one (*ENST00000177694*) has consistently higher expression than the other (*ENST00000581328*). The first also has a higher APPRIS score (PRINCIPAL:1) and is also selected as the dominant isoform by us. Similar considerations hold for (among other) *IL18R1*, *JAK2*, *ZAP70*, *LCK* (for the latter our analysis is able to pick out as dominant a PRINCIPAL:1 transcript out of 14 splice variants). Even when a gene lacks a principal isoform, abundance alone can be a proxy for dominance, as in the case of *GATA3* (an early regulator of T-cell differentiation), where one of the two isoforms showing high expression, *ENST00000379328* and *ENST00000346208*, is selected as dominant (the first, which happens to coincide with the highest APPRIS score for this gene, a PRINCIPAL:4). For other genes, instead, abundance is not a useful indicator. For instance, for the *ITK* gene (*IL2* inducible tyrosine kinase, expressed in T-cells and playing a role in T_H_ differentiation), none of the nine transcript isoforms that survive our filtering has an expression significantly higher than the others. Nevertheless our algorithm is able to identify the only isoform labeled PRINCIPAL:1 as the dominant isoform for that gene. Another example of the same type is *HMGB1*, whose (equally abundant) multiple transcript variants are said to encode for the same protein (see GeneCards PAGE, accessed April 2021).

Another key feature of our approach is that each Bayesian network can be treated independently, as it corresponds to a model of the protein-coding unit associated with a gene/protein. Hence our >4000 Bayesian networks can be run in parallel, which breaks down the complexity to a manageable size, and enables the systematic in-depth analysis carried out in the article. Of course, in reality, the protein-coding units are coupled, for instance through the sharing of chromatin peaks or the association in protein complexes, as we have analyzed in the Results section, or through the action of transcription factors, through the concentration of polymerases, through the ribosomal machinery, etc. All of these latter factors must however be treated as unmodeled hidden variables, as we lack any specific measurement for their quantification. Some of these factors (most probably TF) are likely responsible for the limited validation percentage of certain types of conditional independencies, in particular for the s−s conditional independences. For this case, for instance, we have managed to show that verification of the s−s conditional independences grows with the number of TF potentially associated with the splices involved. This results may be reflecting the fact that a more (complex and hence) precise regulation is less prone to noisy measures of expression, and hence lead to more clear s−s interactions.

If for s−s conditional independencies the percentage of success is only 33.8%, it grows to 57.9% for a−a conditional independencies. It is worth noticing that none of these two classes lead to modifications of the DAG that are justifiable physically or mathematically. From a physical point of view, having a direct dependence between two *s* or between two *a* does not seem to be a realistic assumption. From a mathematical modeling point of view, s−s and a−a pairs are so-called “moral edges” (see STAR Methods for details on this terminology), and it is known that for Bayesian networks a probability distribution that factorizes over a DAG factorizes also over the graph obtained adding the moral edges to the DAG.

When instead we look at independence relationships between different “layers” (the most important types of conditional independencies when it comes to structure validation), then the percentages of confirmations are significantly higher: 74.1% for a→p conditional independencies, and 97.5% for a→s conditional independencies. The latter are the only independencies that may lead to a straightforward modification of the DAG, meaning that when a conditional independence a→s cannot be confirmed, then the presence of an extra edge a→s in the DAG cannot be ruled out. In summary, our validation analysis of the predicted conditional independencies largely confirms the factorization of the probability density functions determined by the DAGs, and suggests the addition of extra 415 causal dependencies of type a→s, i.e., a mere 2.5% of the already existing edges.

The idea that balance can be of importance in biological networks is not new, and was observed for instance in gene regulatory networks in Alon (2007) under the name of coherence, as well as in other classes of biological networks in Iacono and Altafini (2010); Sontag (2007); Torres and Altafini (2016). The role it plays for our Bayesian networks is however novel to the best of the authors’ knowledge. It is related to the already mentioned facts of working in a over-constrained regime (more data than degrees of freedom) and of making use of a symmetric similarity measure, like correlation. In fact, *R* symmetric leads to the presence of cycles in G(R), whose nodes are all experimentally sampled. When evaluated on a cycle, the sample correlations provide an intrinsic test of consistency for the values of the variables involved in the cycle.

To understand the origin of this consistency, it is useful to look at the smallest possible nontrivial correlation graph, a triangle, formed by the variables x,y, and *z* obtained from a fork or chain motif in a DAG (see Figure 1C), and yielding a 3×3 correlation matrix *R*. When G(R) is balanced, the patterns of signs of $R_{xy}$, $R_{xz}$ and $R_{yz}$ are always compatible with each other, and never contradict each other. For instance, if *x* and *y* are negatively correlated and so are *y* and *z*, then *x* and *z* must be positively correlated (in structural balance theory: “the enemy of my enemy is my friend”, see Facchetti et al. (2011)). Internal consistency in a triangle of correlations means that the empirical trends we have obtained from our data for *x*, *y*, and *z* confirm each other. On the other hand, on an unbalanced cycle the opposite happens: the data for *x*, *y* and *z* are guaranteed to contradict each other. One consequence of such inconsistency is that the data become unreliable when used for learning (or confirming) conditional independences, meaning that balance gives us a way to label certain interaction patterns as “more plausible” or as “less plausible” based on the samples. The generalization from a triangle to any arbitrary correlation graph (and to a concentration graph) can be done along similar conceptual lines.

To understand the mechanism by which balance and inverse balance lead to confirmation of conditional independencies in such a systematic way, it is useful to observe what happens to the correlation between *x* and *y* upon conditioning. If we compare $R_{xy}$ and $R_{xy.C}$, then we see that for most balanced sample correlations graphs it is

(Equation 1) $$\left|R_{xy.C}\right|\leq \left|R_{xy}\right|,$$

meaning that conditioning on a balanced graph normally leads to a contraction, that reduces the dependency between the variables *x* and *y*, see Figure 6A. The contrary often (but not always) happens on unbalanced G(R). This contraction property can be made intuitively clear if we look at the formula for partial correlations on a triangle

$$R_{xy.z}=\frac{R_{xy}-R_{xz}R_{yz}}{\sqrt{1-R_{xz}^{2}}\sqrt{1-R_{yz}^{2}}}.$$

For all possible sign combinations in a balanced triplet, the second term of the numerator has opposite sign to the first term, meaning that in most cases $R_{xy.z}$ obeys to the heuristic (1). There are however exceptions, as can be seen in the density plots of Figure 6B. In the case of inverse balance, the contraction law (1) is obeyed systematically. For covariances, the equivalent of (1) can be shown to be rigorously true, see STAR Methods.

The consistency of signs in a balance graph can be interpreted as solving a system of equations over a binary field, with constraints given by the signs. In statistical physics, it is well-known that checking balance (i.e., checking the absence of frustration in a signed graph) can be mapped into a so-called XORSAT constraint satisfaction problem, see Facchetti et al. (2011); Mezard and Montanari (2009). Such problem either has a unique solution, when the graph is balanced, or has no solution at all, when the graph is unbalanced. The test is akin to a “parity check” used in error-correcting codes in information theory (Cover and Thomas, 2006). What is novel in our approach is the observation that balance and inverse balance provide good heuristics for reduction of correlation upon conditioning, and hence for verification of conditional independencies.

While such tests are in our knowledge novel for balance, somewhat related ideas have been developed in the field of Gaussian graphical models for inverse balance. In particular in Lauritzen et al. (2019); Slawski and Hein (2015), it is shown how a (signed) MTP_2_ distribution (see STAR Methods) imposes constraints on the possible sign pattern of the correlation matrix, thereby allowing to reconstruct such matrix even when data on some of the variables are missing. The underlying principle in this literature is the following: a property of the multivariate joint probability distribution provides constraints that must be obeyed also by the empirical sample probability distribution if the latter is indeed drawn from the former.

The underlying principle in our approach is instead the following: a sample probability distribution provides constraint for its own consistency. These constraints do not require us to make any assumption on the “true” multivariate joint probability distribution from which the data are drawn. The constraints are nevertheless useful in practice in situations in which all variables are measured, as they provide a sort of “parity check” on the sampled data. We expect this type of reasoning to be applicable also in other contexts in which Bayesian networks are of relevance.

### Limitations of the study

Several limitations have already been discussed above, notably the fact that the protein coding units are in reality not independent, but coupled by unmeasured quantities (TF, shared transcriptional/translational machinery etc.). Another limitation comes from the fact that the data we have available correspond to time series, for which the assumption of stationarity is not rigorously true. Using dynamical correlation partially, but not completely, eliminates the issue. Other potential limitations come from the data we are using, in particular the fact that mass-spec is not sensitive enough to detect protein isoforms is a significant bottleneck of the current study. Similarly, the uncertainty in how the RNA sequences are assigned to the various splice variants of a gene is of great relevance in our approach, as it may lead to a bias in the analysis.

## STAR★Methods

### Key resources table

**Table:** 

REAGENT or RESOURCE	SOURCE	IDENTIFIER
**Deposited data**		

RNA-seq data	Magnusson et al., 2020	E-MTAB-7775
Mass-spectrometry data	Magnusson et al., 2020	PXD013361
ATAC-seq data	Rundquist et al., 2022	E-MAT-10444

**Software and algorithms**		

MatLab	MathWorks	https://www.mathworks.com/login

### Resource availability

#### Lead contact

Further questions should be directed to the lead contact, Prof. Claudio Altafini (claudio.altafini@liu.se).

#### Materials availability

This study did not generate new data.

### Methods details

#### Isolation of CD4^+^ T helper (T_H_) cells and T_H_1 polarization

Peripheral blood mononuclear cells (PBMC) were isolated from blood donor derived buffy coats through gradient centrifugation (Lymphoprep, Axis shields diagnostics, Dundee, Scotland). Naive CD45RA + CD4^+^T-cells were subsequently isolated with magnetic bead separation using the “Naive CD4^+^T-cell Isolation Kit II, human” (Miltenyi Biotec, Bergisch Gladbach, Germany). The cells were then activated and polarized toward T_H_1 using Dynabeads™. Human T-Activator CD3/CD28 (1 bead/cell) (Dynal AS, Lillestøm, Norway), 5 ng/μl recombinant human IL-12p70, 10 ng/μl recombinant human IL-2 and 5 μg/μl anti-IL-4 antibodies (clone MAB204) (all three from, Bio-Techne, Minneapolis, USA), in RPMI 1640 media (Gibco, Paisley, United Kingdom). A portion of T-cells used for RNA and protein isolation was obtained at baseline and after 0.5 h (only RNA), 1 h, 2 h, 6 h, 24 h and 5 days (only protein; point not used in the analysis). The cells were washed twice in PBS, snap frozen in a dry ice ethanol bath and stored at −80°C until use. During the protein and RNA extractions, multiple samples were pooled from twelve different individuals to reach the necessary amount of material for the subsequent analysis steps.

#### RNA-seq sample preparation

RNA was isolated using a ZR-Duet DNA/RNA kit (Zymo Research, Irvine, USA) and stored at −80°C. RNA library preparation and the subsequent RNA-sequencing were carried out by the Beijing Genomics Institute (https://www.bgi.com/global/). Library preparation was performed using the TruSeq RNA Library Prep Kit v2 (Illumina, San Diego, USA). Each sample was sequenced to the depth of 40 million reads per samples with pair end sequencing and a read length of 100bp on an Illumina 2500 instrument.

#### ATAC-seq sample preparation

The ATAC.seq sample preparation was carried out in accordance with the OMNI-ATAC-seq protocol in Corces et al. (2017), modified to incorporate the Nextera tagmentation reagents (Zuberbuehler et al., 2019).

#### Isolation of CD4^+^ T helper cells and T_H_1 polarization

Naïve T-cells were isolated and differentiated towards T_H_1 for ATAC-seq as described above and sampled at the time points of 0.5, 1, 2, 6 and 24 h (same as the RNA-seq). The naïve T-cells were differentiated on a 96-well plate with 50,000 cells per differentiation reaction (each time points was its own differentiated reaction), in biological triplicate. All time points were from the same individual for each replicate.

#### Nuclei extraction

For nuclear extraction, at each time point the cells were transfer to DNA low-binding tubes (Eppendorf, Hamburg, Germany) and washed with 1ml PBS by centrifugation at 500g for 5 min. After centrifugation the supernatant was discarded by pipette and the cells resuspended in 50 μl ATAC-seq RSB buffer (10 mM Tris-HCl pH 7.4, 10 mM NaCl and 3 mM MgCl2) containing 0.1% NP40, 0.1% Tween-20 (all from Sigma-Aldrich, St. Louis, MO) and 0.01% digitonin (Promega, Madison, WI) and incubated for 3 min. Then, 1 ml ATAC-seq RSB containing 0.1% Tween 20 was added and the nuclei were pelleted by centrifugation at 500*g* for 10 min and the supernatant discarded. All steps for the nuclei extraction were carried out on ice and all buffers were pre-cooled to 4°C.

#### Sequencing library preparation and sequencing

ATAC-seq library preparation was carried out immediately by resuspending the nuclei pellet in 50 μl transposition mix (25 ml 2X TD buffer, 2.5 μl transposase (both from Illumina, San Diego, CA), 16.5 μl PBS, 0.5 μl digitonin, 0.5 μl 10% Twenn-20 and 5 ml Milli-Q water). The reaction mix was then incubated for 30 min at 37°C in a Eppendorf themomixer comfort (Eppendorf, Hamburg, Germany) at 1000 rpm and the reaction subsequently stopped by the addition of 250 μl DNA binding buffer from a Zymo-clean and concentrator kit (Zymo-Research, Irvine, CA) and the DNA purified using said Zymo kit. The ATAC-seq DNA libraries were then amplified and indexed with a NEBNext index kit (NEB, Ipswich, MA) by PCR with the following cycling conditions: 98°C 30 s, 72°C 5 min, followed by 5 cycles of 98°C 10 s, 63°C 30 s, 72°C 1 min. Following PCR DNA concentration was assayed by qPCR as described in Buenrostro et al. (2015) and additional PCR cycles were added as necessary. After PCR the samples were once again purified using the Zymo-clean and concentrator kit. Library quality was assessed by fluorimetry with a Qubit dsDNA HS Assay Kit (Thermo Fisher Scientific, Waltham, MA) and gel electrophoresis using a Fragment Analyzer (Thermofisher Scientific). Sequencing of the libraries was carried out on an Illumina NovaSeq 6000 instrument by the National Genomics Infrastructure of Sweden (https://www.scilifelab.se/facilities/ngi/).

#### Chemicals

Urea, Ammonium bicarbonate (ABC), Triethylammonium bicarbonate (TEAB), Iodoacetamide (IAA), Ammonium formate, Trifluoroacetic acid (TFA) and formic acid were purchased from Sigma-Aldrich (St. Louis, MO). Dithiothreitol (DTT) were purchased from Roche (Hoffmann, Switzerland). Protein assay kit (# 23235) and TMT 6 plex isobaric labeling kits were purchased from Thermo Scientific (MA, USA) and MS grade Trypsin was purchased from Promega (Madison, WI).

#### Cell lysis

The isolated cells from patients were resuspended in 100 μl of 8 M Urea in 40 mM Tris-HCl (pH 7.6) and pooled with a similar number of total cells between biological replicates. The suspension was sonicated using focus sonicator (Sonic Dismembrator 500, Fisher Scientific) for 3 cycles of 10 s pulse with 10 s intervals at 10% of power. After sonication, a magnetic rack was used to remove the T-Activator beads used for the polarization. Protein concentration was measured using BCA assay and 40 μg of each sample was digested with trypsin using In-solution digestion.

#### In solution digestion

First reduction and alkylation of disulfide bonds on proteins were carried out using 1 M DTT, final sample concentration 10 mM, for 45 min and 1 M IAA, final sample concentration 30 mM, for 30 min in the dark, respectively. Following alkylation and reduction the samples were diluted with ammonium bicarbonate buffer (pH 8.0) until the urea concentration was 1 M. The proteins were digested with trypsin overnight at 37°C at an enzyme to protein ratio of 1:20. Finally, the peptides were acidified with 100% trifluoroacetic acid (TFA) to a final concentration of 1% TFA and then desalted using macro spin columns (Harvard apparatus, USA).

#### TMT labeling

Peptides were labeled with 6-plex TMT reagent using manufacturer’s protocol with some modification. The six peptide samples from each time series was resuspended in 100 μl of 100 mM TEAB buffer (pH 8.0) and a unit of each TMT reagent was resuspended in 40 μl of acetonitrile. Subsequently, the prepared TMT reagent was transferred to the peptide sample and then vortexed. Next, the samples were incubated for 2 h at room temperatures. Finally, all of the labeled peptide samples from each time series were pooled and concentrated by vacuum centrifugation. The labeled sample was resuspended 100 μl with 10 mM ammonium formate in water (pH 10) and subjected to high-pH reverse-phase liquid chromatography fractionation.

#### High pH fractionation

The TMT labeled peptides were fractionated into 24 fractions. Peptide samples were separated using an analytical column (Xbridge, C18, 5 μm, 4.6 mm × 250 mm) on the Agilent 1200 series HPLC system. Peptides were eluted using following gradient over 115 min: 0–10 min, 0% B, 10–20 min, 5% B, 20–80 min, 35% B, 80–95 min, 70% B, 95–105 min, 70% B, 105–115 min, 0% B; 10 mM ammonium formate (pH 10) was mobile phase A, and 10 mM ammonium formate with 90% ACN (pH 10) was mobile phase B. The 96 fractions were added up into 24 fractions by combining Fraction 1 - Fraction 25 - Fraction 49 - Fraction 73, Fraction 2 - Fraction 26 - Fraction 50 - Fraction 74 … Fraction 24 - Fraction 48 - Fraction 72 - Fraction 96. All fractions were vacuum dried and stored at −80°C after the desalting step.

#### LC-MS analysis

The fractionated peptides were analyzed on an Orbitrap Fusion Lumos Tribrid Mass Spectrometer (source) coupled with the Easy-nLC 1200 nano-flow liquid chromatography system (Thermo Fisher Scientific). The peptides from each fraction were reconstituted in 0.1% formic acid and loaded on an Acclaim PepMap100 Nano-Trap Column (100 μm × 2cm, Thermo Fisher Scientific) packed with 5 μm C18 particles at a flow rate of 5μl per minute. Peptides were resolved at 250-nl/min flow rate using a linear gradient of 10%–35% solvent B (0.1% formic acid in 95% acetonitrile) over 95 min on an EASY-Spray column (50 cm × 75 μm ID), PepMap RSLC C18 and 2 μm C18 particles (Thermo Fisher Scientific), which was fitted with an EASY-Spray ion source that was operated at a voltage of 2.3 kV. Mass spectrometry analysis was carried out in a data-dependent manner with a full scan in the mass-to-charge ratio (m/z) range of 350 to 1,800 in the “Top Speed” setting, 3 s per cycle. MS1 and MS2 were acquired for the precursor ions and the peptide fragmentation ions, respectively. MS1 scans were measured at a resolution of 120,000 at an m/z of 200. MS2 scan was acquired by fragmenting precursor ions using the higher-energy collisional dissociation method and detected at a mass resolution of 30,000, at an m/z of 200. Automatic gain control for MS1 was set to one million ions and for MS2 was set to 0.1 million ions. A maximum ion injection time was set to 50 ms for MS1 and 100 ms for MS2. Higher-energy collisional dissociation was set to 35 for MS2. Precursor isolation window was set to 0.7 m/z. Dynamic exclusion was set to 35 s, and singly-charged ions were rejected. Internal calibration was carried out using the lock mass.

#### Peptide and protein identification

The obtained data were analyzed using MaxQuant (v 1.6.0.1). MS raw data were searched using Andromeda algorithm with matching to the Uniprot human reference (released in Nov, 2017). A specificity of trypsin was determined at up to 2 missed cleavages. In modification, carbamidomethylation, TMT 6-plex modification at lysine and N-termination were set as the fixed modifications, and oxidation of methionine was set as a variable modification. The false discovery rate (FDR) for peptide level was evaluated to 0.01 for removing false positive data. For highly confident quantifications of protein, protein ratios were calculated from two or more unique quantitative peptides in each replicate. Data was normalized and removed contaminant and razor peptide. To enrich differentially expressed proteins (DE-Ps), we analyzed the quantitative ratios (as the Log2 value). The fold-change ratio cut off was more than 2 or less than 0.5 based on intensity of unstimulated sample. Searched data went through statistical process with Perseus (v 1.5.1.6).

#### RNA-seq data processing

The RNA-seq data was processed using the following pipeline. Sample qualities were assessed with fastQC and the mRNA reads were subsequently aligned using STAR Dobin et al. (2013) to the “Homo_sapiens.GRCh38.dna.primary_assembly.fa” from Ensembl. The resulting read alignment bam files were assembled into transcripts with StringTie Pertea et al. (2015) using the GRCh38.90 gtf annotation from Ensembl. mRNA Transcripts were mapped to the mass spectrometry signal of protein abundance using the Homo.sapiens and Mus.musculus package in R.

#### ATAC-seq data processing

General sample qualities were assessed as for the RNA-seq with fastQC. Transcriptional Start Site (TSS) enrichment for the samples were calculated with tssenrich (https://pypi.org/project/tssenrich/) and found to be acceptable at an average value of 5 (see https://www.encodeproject.org/atac-seq/ for ATAC-seq quality standards). Sequencing adapters were trimmed using Trim-Galore (https://github.com/FelixKrueger/TrimGalore), and the reads aligned to the “GCA_000001405.15_GRCh38_no_alt_analysis_set.fna” from Refseq using Bowtie2 (Langmead and Salzberg, 2012). ATAC-seq signal peaks were called using “Genrich” (https://github.com/jsh58/Genrich) and reads in peaks counted with “featurecounts” from the Rsubread package (Liao et al., 2019).Prior to counting of reads in peaks duplicate reads were dropped with “Picard MarkDuplicates” and all alignments shifted with “alignmentSieve –ATACshift” from deeptools (Ramirez et al., 2016). Peaks were then assigned to the promoter of transcripts with a flanking transcript distance of 5000bp, see Yu et al. (2015), by mapping the peaks to the promoter of transcripts in the GRCh38.90 gtf annotation from Ensembl, performed in python. Promoters were defined as ±3000bp from their TSS. This threshold is similar to the values found in the literature (Corces et al., 2016; Fullard et al., 2018; Wu et al., 2018).

#### Data filtering and analysis

Our datasets consist of 41466 ATAC-seq time series, 147119 RNA-seq time series (of which 49890 are labeled as “protein coding”splice variants according to Ensembl Hg38) and 6909 proteins mass-spec time series. The time series for ATAC-seq and RNA-seq consist of the following time points (in hours): $t_{a}=t_{s}=\left[0\;0.5\;1\;2\;6\;24\right]\;h$ , while for the protein data$t_{p}=\left[0\;1\;2\;6\;24\;120\right]\;h$. The t=120h time point is disregarded in the following. All time series consist of 3 biological replicates. The data were interpolated using cubic splines, to a resolution of 0.5h, meaning that after interpolation we have time series of 49 time points. For 4797 of these proteins there exist an association with at least one ATAC-seq peak and a splice variant, hence we have 4797 ‘protein-coding units’ represented as DAGs. These DAGs involve 8110 ATAC-seq peaks and 27642 alternative splice variants, the latter after filtering for the “protein coding” annotation in the Ensembl database, and after excluding time series missing too many points or not linked to any ATAC-seq peak.

#### Functional annotation for splice variants of a gene

In the APPRIS database (Rodriguez et al., 2018), splice variants of a gene are labelled as PRINCIPAL:ξ, where ξ is an integer running between 1 and 5 according to importance (1= most important), ALTERNATIVE:ξ, with ξ=1,2, and MINOR. Other splice variants irrelevant for translation (retained introns, processed transcripts, nonsense mediated decay, non-coding transcripts, etc.) are here classified as NON-CODING. A few splice variants for which an APPRIS classification is missing are denoted as ABSENT. For the 27642 splice variants present in our DAGs, the distribution of APPRIS scores is shown in Figure 4A of the article, yellow bars. In particular, around 62% of the 4797 DAGs have one splice which is labeled as PRINCIPAL:1 in APPRIS (see violet bars in Figure 4A).

#### Compiling a TF - splice variant interaction map

A map of TF - binding sites was assembled, involving 656 TF and a total of around 6.5M potential interactions with our 27,642 transcript isoforms. This was achieved in three steps. A chromatin peak - splice variant interaction map results from the association procedure described above. ATAC-seq can also be used to infer which TFs regulate gene transcription. In fact, TFs binding events leave behind a characteristic mark: the sequence of DNA where the binding occurred presents a drop in the accessibility—these sequences are usually called *footprints*. By scanning each footprint for known TF motifs, using a TF binding site database (HOCOMOCO v.11, see Kulakovskiy et al. (2018)), we have associated each peak to a list of potential TFs. This constitutes the second interaction map, TF-peak.

Lastly, we have combined the two interaction maps (i.e. peak-splice variant and TF-peak) to obtain a TF-splice variant interaction map.

#### Sample dynamical correlation

Let the vector x(t) represent ATAC-seq, RNA-seq or mass-spec data, measured at a finite number of time points $t_{1}<t_{2}<\ldots <t_{N}$. The method to compute sample dynamical correlations described in Opgen-Rhein and Strimmer (2006) is based on computing a numerical approximation of the inner product

(Equation 2) $$\left\langle x_{i}\left(t\right),x_{j}\left(t\right)\right\rangle =\frac{1}{t_{N}-t_{1}}\int_{t_{1}}^{t_{N}}x_{i}\left(t\right)x_{j}\left(t\right)\mathrm{d}t$$

where$x_{i}\left(t\right)$ and $x_{j}\left(t\right)$ are components of x(t). To calculate (2) we assume $x_{i}\left(t\right)$ and $x_{j}\left(t\right)$ follow a linear approximation between the sampling time points, yielding

$$\left\langle x_{i}\left(t\right),x_{j}\left(t\right)\right\rangle =\overset{N}{\underset{k=1}{\sum }}x_{i}\left(t_{k}\right)x_{j}\left(t_{k}\right)\frac{t_{k+1}-t_{k-1}}{2\left(t_{N}-t_{1}\right)},$$

where $t_{0}=t_{N+1}=0$. Consider the averages over time of $x_{i}$and $x_{j}$, indicated with ${\bar{x}}_{i}=\frac{1}{N}\sum_{k=1}^{N}x_{i}\left(t_{k}\right)$ and ${\bar{x}}_{j}=\frac{1}{N}\sum_{k=1}^{N}x_{j}\left(t_{k}\right)$, and the centered functions $x_{i}^{C}=x_{i}-{\bar{x}}_{i}$ and $x_{j}^{C}=x_{j}-{\bar{x}}_{j}$. The sample dynamical covariance can be obtained by taking the inner product between the centered functions $x_{i}^{C}\left(t\right)$ and $x_{j}^{C}\left(t\right)$

$${\text{Σ}}_{x_{i}x_{j}}=\left\langle x_{i}^{C}\left(t\right),x_{j}^{C}\left(t\right)\right\rangle ,$$

while the corresponding sample dynamical correlation is

$$R_{x_{i}x_{j}}=\left\langle x_{i}^{S}\left(t\right),x_{j}^{S}\left(t\right)\right\rangle$$

where $x_{i}^{S}\left(t\right)=x_{i}^{C}\left(t\right)/\sqrt{\text{Var}\left[x_{i}\left(t\right)\right]}$ and $x_{j}^{S}\left(t\right)=x_{j}^{C}\left(t\right)/\sqrt{\text{Var}\left[x_{j}\left(t\right)\right]}$ are the standardized functions and $\text{Var}\left[x_{i}\left(t\right)\right]=\left\langle x_{i}^{C},x_{i}^{C}\right\rangle$, $\text{Var}\left[x_{j}\left(t\right)\right]=\left\langle x_{j}^{C},x_{j}^{C}\right\rangle$ the corresponding sample variances. Intuitively, this definition assigns a positive (negative) correlation coefficient to time-series that appear mostly on the same (opposite) side of their time average.

In general, experiments are repeated multiple times, obtaining *M* replicates of each realization. We will denote $x_{ik}\left(t\right)$, k∈1,…,M, the *k*-th replicate of $x_{i}\left(t\right)$. In order to compute the dynamical correlation, as before we start by calculating for each replicate $x_{ik}\left(t\right)$ and $x_{jk}\left(t\right)$ their time averages $\bar{x_{ik}}$ and $\bar{x_{jk}}$. This leads to the centered functions $x_{ik}^{C}\left(t\right)=x_{ik}\left(t\right)-\frac{1}{M}\sum_{r=1}^{M}\bar{x_{ik}}$ and $x_{jk}^{C}\left(t\right)=x_{jk}\left(t\right)-\frac{1}{M}\sum_{r=1}^{M}\bar{x_{jk}}$ and the variance estimates

$$\begin{array}{l} \text{Var}\left[x_{ik}^{C}\left(t\right)\right]=\frac{1}{M-1}\overset{M}{\underset{r=1}{\sum }}\left\langle x_{ik}^{C}\left(t\right),x_{ik}^{C}\left(t\right)\right\rangle \\ \text{Var}\left[x_{jk}^{C}\left(t\right)\right]=\frac{1}{M-1}\overset{M}{\underset{r=1}{\sum }}\left\langle x_{jk}^{C}\left(t\right),x_{jk}^{C}\left(t\right)\right\rangle . \end{array}$$

Finally, the standardized functions $x_{ik}^{S}\left(t\right)=x_{ik}^{C}\left(t\right)/\sqrt{\text{Var}\left[x_{ik}\left(t\right)\right]}$ and $x_{jk}^{S}\left(t\right)=x_{jk}^{C}\left(t\right)/\sqrt{\text{Var}\left[x_{jk}\left(t\right)\right]}$ can be used to estimate the sample dynamical correlation

$$R_{x_{i}x_{j}}=\frac{1}{M-1}\overset{M}{\underset{r=1}{\sum }}\left\langle x_{ik}^{S}\left(t\right),x_{jk}^{S}\left(t\right)\right\rangle .$$

The corresponding sample dynamical covariance is of course given by

$${\text{Σ}}_{x_{i}x_{j}}=\frac{1}{M-1}\overset{M}{\underset{r=1}{\sum }}\left\langle x_{ik}^{C}\left(t\right),x_{jk}^{C}\left(t\right)\right\rangle .$$

#### A normality test for detrended data

As mentioned above, the data for ATAC-seq, RNA-seq and mass-spec correspond to time series, and do not directly obey an assumption of stationarity nor of Gaussianity. The situation is however different for the detrended data $x_{ik}^{S}$ described in the previous section. To check it, we used a Kolmogoron-Smirnov normality test, with p value equal to 0.01. For ATAC-seq, none of the 8110 data series can reject the null hypothesis of normality, while for the detrended data only 855 (i.e., 10.5%) can still reject the normality null hypothesis. For RNA-seq, the corresponding numbers are 27,628 out of 27642 for the original data, and 9176 (i.e., 33.2%) for the detrended data. Finally, for the mass-spec data, if none of the 4797 original data series can reject the normality null hypothesis, only 18 (i.e., 0.4%) can still reject it after detrending.

#### Protein-coding units as Bayesian networks

To each protein we can associate a protein-coding unit formed by the alternative splice variants of the gene coding for that protein (according to the Ensembl classification, database build Hg38, Cunningham et al. (2018)), and by the chromatin peaks associated to those splice variants. The protein-coding unit represents the largest possible graph associated with the synthesis of a given protein for the data we have available. Let a,s, and *p* denote the variables representing resp. chromatin site accessibility, splice variant expression, and protein abundance. The causal dependencies we consider are a→s and s→p. Physically, these edges are unambiguously directed, meaning that all paths of the graph are directed and of length at most 2 (a→s→p). The graph is therefore a 3-layer DAG, and typically it looks like in Figure 1A. For each protein, the corresponding DAG can be modeled as a Bayesian network with continuous random variables associated to the nodes and joint probability distribution that factorizes into a product of conditional probabilities according to the DAG. Overall, from our datasets we have 4797 DAGs of size that varies from 3 to 52 nodes, see Figure 3A. Different protein-coding units involve different genes and proteins, while for chromatin accessibility only a small fraction of peaks (8%) is shared by two or more genes. Since the node sets of different DAGs are essentially non-overlapping, each DAG can be treated independently, leading to a massively parallel set of Bayesian networks.

#### Computing conditional independencies

Roughly speaking, in Bayesian networks missing edges on a DAG correspond to pairs of variables that are either independent or conditionally independent given some other variables, see e.g. Koller and Friedman (2009). Let *x* and *y* be two variables corresponding to nodes of a DAG (i.e., *x* and *y* are any of the *a*, *s* and *p* localized on a protein-coding unit). Independence between *x* and *y* is indicated x⊥y, while conditional independence given a set C of variables is denoted x⊥y|C. For instance, choosing C=pa(x), the set of parent nodes of *x*, Bayesian network theory predicts that $x\;⊥\;y|\text{pa}\left(x\right)$ whenever *y* is a non-descendent of *x*. These conditions are called local conditional independencies (or local Markov conditions). In general, the local Markov conditions do not exhaust all conditional independencies encoded in a Bayesian network. Other, less straightforward, independencies are obtained by checking the so-called d-separation property, see Pearl (1988). The resulting conditional independencies are referred to as global Markov conditions: x⊥y|S, where C=S is a d-separation set for the pair *x* and *y*. For the class of DAGs discussed in this article, consisting of 3 clearly defined layers (*a*, *s*, and *p*) and having edges only between adjacent layers (a→s and s→p), it is always possible to order the nodes so that local and global Markov conditions coincide (Koller and Friedman, 2009; Lauritzen, 1996). These conditional independencies can be systematically computed from the topology of our DAGs. Overall in the 4797 DAGs considered in the article there are more than $1.5\;\cdot \;10^{5}$ conditional independencies, split into four4 categories, a→s, a→p, a−a, s−s, see Table 1. A fifth possible category, s→p, is empty, i.e., *s* and *p* are never considered conditionally independent, as by construction all *s* in a DAG are connected with the corresponding protein *p* (i.e., all *s* are “causing”*p*).

#### A correlation-based verification test for conditional independencies

The conditional independencies computed on the DAGs can be validated empirically via a correlation analysis. Since our time series represent a cellular dynamical process (cell differentiation), we decided to use the dynamical correlation of Opgen-Rhein and Strimmer (2006) in place of ordinary correlation, because it can account for longitudinal data that are not stationary, as observed above. In the article we always omit the adjective “dynamical” when mentioning correlations and partial correlations.

Given *x* and *y* colocalized on the same DAG, let $R_{xy}$ be the corresponding sample correlation. We say that the independence condition x⊥y is verified (or validated) by the data if $|R_{xy}|<\theta$, where θ is the threshold provided by a Fisher z-test. Similarly, the conditional independence condition x⊥y|C is verified by the data if $|R_{xy.C}|<\theta$, where $R_{xy.C}$ is the partial correlation between *x* and *y* conditioned on C.

#### Bayesian networks and conditional independence

A Bayesian network is a probabilistic graphical model on a Directed Acyclic Graph (DAG) in which to each node is associated a random variable $X_{i}$. The joint probability distribution that represents the DAG factorizes according to the structure of the DAG. For instance, for the DAG of Figure 1A of the article, of variables $X_{i}=\left\{a_{1},\;a_{2},\;s_{1},\;s_{2},\;s_{3},p\right\}$, we have

$$P\left(a_{1},\;a_{2},\;s_{1},\;s_{2},\;s_{3},p\right)=P\left(a_{1}\right)P\left(a_{2}\right)P\left(s_{1}|a_{1}\right)P\left(s_{2}|a_{2}\right)P\left(s_{3}|a_{2}\right)P\left(p|s_{1},s_{2},s_{3}\right).$$

The topology of the DAG defines also a series of *conditional independence* relationships which are normally classified into local and global Markov independencies, see Koller and Friedman (2009). Local Markov conditions are expressed as

$$X_{i}\;⊥\;X_{j}|\text{pa}\left(X_{i}\right)$$

where$\text{pa}\left(X_{i}\right)$ are the parents of $X_{i}$, and $X_{j}$ is a non-descendent node of $X_{i}$. Global Markov conditions are instead obtained computing the so-called d-separation set (Pearl, 1988), S, i.e., the set isolating $X_{i}$ from the rest of the DAG: if $X_{j}\notin S$, then

$$X_{i}\;⊥\;X_{j}|S.$$

If the nodes of the DAG are “well-ordered”, i.e., if a topological order is chosen so that $X_{i}\in \text{pa}\left(X_{j}\right)$ implies i<j, then local and global Markov conditions coincide. For instance, for the DAG in Figure 1A of the article the Markov conditions are:

$$\begin{array}{l} a_{1}\;⊥\;a_{2},\;s_{1}⊥\;s_{2}|a_{1},a_{2},\;s_{1}\;⊥\;s_{3}|a_{1},a_{2},\;s_{2}\;⊥\;s_{3}|a_{2},\\ a_{1}⊥\;p|s_{1},\;a_{2}⊥\;p|s_{2},s_{3},\;s_{1}⊥\;a_{2}|a_{1},\;s_{2}\;⊥\;a_{1}|a_{2},\;s_{3}\;⊥\;a_{1}|a_{2} \end{array}$$

where some of the conditioning sets can be reduced (e.g., $s_{1}⊥\;s_{2}|a_{1},a_{2}$ can be written as $s_{1}\;⊥\;s_{2}$, etc.).

#### Moral edges and moral graphs

Starting from a DAG D, one can construct the so-called moral graph $D^{m}$ obtained by adding to D the undirected edges connecting co-parents of a node (called moral edges), and dropping all edge arrows. By construction, for our DAGs, all s−s edges are moral edges because all splice variants are connected to the corresponding protein *p*, and the motif formed by $s_{1}$ and $s_{2}$ with *p* is always a so-called *v*-structure $s_{1}\to p\leftarrow s_{2}$. The corresponding d-separating set between $s_{1}$ and $s_{2}$ never involves *p*, but only upstream causes (i.e., one or more *a*, see example on the left in Figure 1C). Similar moral edges can occur also when two *a* are connected to the same splice variant *s*. From the topology of the DAGs, these can only be associated to marginal independencies, not to conditional independencies. Not all a−a independencies we consider are however moral edges, see e.g. Figure 1A. None of these s−s or a−a edges is part of the original DAG D, but they are of $D^{m}$. However, if a probability distribution factorizes over D it factorizes also over $D^{m}$. Hence the conditional independencies associated to moral edges s−s or a−a can be considered of “secondary importance” for what concerns the determination of the causal dependency structure among the variables of a DAG.

#### Gaussian graphical models

The probability density function of a *n*-dimensional Gaussian distribution over $X=\left\{X_{1},\ldots ,X_{n}\right\}$ (denoted X in vector form) is given by

$$f\left(X\right)=\frac{1}{{\left(2\pi \right)}^{n/2}|\text{Σ}{|}^{1/2}}\text{exp}\left(-\frac{1}{2}{\left(X-\mu \right)}^{T}{\text{Σ}}^{-1}\left(X-\mu \right)\right)$$

where X∼N(μ,Σ). From now on we assume that μ=0, and that the covariance matrix Σ is symmetric, positive definite (p.d.). $K={\text{Σ}}^{-1}$is called the concentration matrix (or information matrix, or precision matrix) of the multivariable distribution and is also a p.d. matrix. Just like the correlation matrix *R* is the normalization of Σ by the diagonal matrix $\Delta_{1}=\text{diag}\left(\sqrt{{\text{Σ}}_{11}},\ldots ,\sqrt{{\text{Σ}}_{nn}}\right)$, i.e., $R=\Delta_{1}^{-1}\sum \Delta_{1}^{-1}$, so we can consider the normalization of *K* by $\Delta_{2}=\text{diag}\left(\sqrt{K_{11}},\ldots ,\sqrt{K_{nn}}\right)$: $H:=\Delta_{2}^{-1}K\Delta_{2}^{-1}$. Straightforward calculations give that $H=\Delta_{3}^{-1}R^{-1}\Delta_{3}^{-1}$where $\Delta_{3}=\Delta_{1}\Delta_{2}$. Both *R* and *H* are obviously p.d. and have the same sign pattern as Σ resp. *K*. Notice that in the article, for simplicity of notation, we use a concentration matrix $J=R^{-1}$, which of course corresponds to $J=\Delta_{3}H\Delta_{3}$.

The partial correlation between the variables $X_{i}$ and $X_{j}$ given the remaining n−2 variables $X^{c}=X\setminus \left\{X_{i},X_{j}\right\}$ is defined as

$$R_{ij.X^{c}}:=-\frac{{\left(R^{-1}\right)}_{ij}}{\sqrt{{\left(R^{-1}\right)}_{ii}{\left(R^{-1}\right)}_{jj}}},$$

or, in matrix form, P=2I−H. This formula shows that the concentration matrix *H* and the partial correlation matrix *P* have the same nonzero pattern but with opposite signs. *P* is in general not p.d.

#### Balance and inverse balance on Bayesian networks

Consider the graph G(R) formed by taking the correlation matrix *R* as adjacency matrix. Because, in general, R≶0, G(R) is a signed graph. The signed graph G(R) is said to be *balanced* (or *structurally balanced*, see Facchetti et al. (2011), or *frustration-free* in statistical physics language, see Mezard et al. (1986); Mezard and Montanari (2009)), if all cycles of G(R) have positive sign, i.e., they contain an even number of negative edges. Denote V the node set of G(R). An equivalent condition to balance is that there exists a partition of V into 2 sets $V_{1}$, $V_{2}$ such that $V_{1}\cap V_{2}=\emptyset$ and $V_{1}\cup V_{2}=V$, for which$R_{ij}\geq 0$ for all $v_{i},v_{j}\in V_{k}$ and $R_{ij}\leq 0$ for all $v_{i}\in V_{k}$ and $v_{j}\in V_{\ell }$, k≠ℓ. Another equivalent condition is that there exists a diagonal signature matrix $D=\text{diag}\left(d_{1},\ldots ,d_{n}\right)$ with $d_{i}=\pm 1$, such that, after the change of basis with $D=D^{-1}$ (also called a gauge transformation), $R_{D}=DRD$ is nonnegative, and therefore $G\left(R_{D}\right)$ has all nonnegative edges. For Gaussian variables $X_{i}$ and $X_{j}$, one says that $X_{i}$ and $X_{j}$ are *positively associated* if for all monotone functions *f* and *g*, $R_{f\left(X_{i}\right)g\left(X_{j}\right)}\geq 0$, which in particular implies that $R_{X_{i}X_{j}}\geq 0$. If a vector of Gaussian random variables X has a balanced correlation graph G(R), then the variables DX are positively associated (they have correlation $R_{D}$). A consequence of positive association is that $X_{i}$ and $X_{j}$ are independent iff $R_{X_{i}X_{j}}=0$ (Thm. 3.7 of Fallat et al. (2017)).

In Gaussian graphical models, a concept stronger than positive association is often used, that of *Multivariate Total Positivity of order 2* (MTP_2_). In terms of the concentration matrix $K={\text{Σ}}^{-1}$ (or of $H={\left({\text{Δ}}_{3}R{\text{Δ}}_{3}\right)}^{-1}$), X∼N(0,Σ) is MTP_2_ if and only if *K* is an M-matrix, i.e., $K_{ij}\leq 0$∀i≠j (and *K* is p.d., hence $K_{ii}>0$). X being MTP_2_ is a sufficient but not necessary condition for X being positive associated (meaning that *K* M-matrix implies Σ[and *R]* nonnegative, see Karlin and Rinott (1983)). In this article, we call *inverse balanced* a Gaussian probability distribution X∼N(0,Σ) such that $K={\text{Σ}}^{-1}$ becomes an M-matrix after a gauge transformation with a diagonal signature matrix *D*, i.e., $K_{D}=DKD$ is an M-matrix. It is straightforward to show that if *D* renders $K_{D}$ nonnegative, then it also renders DRD (and DΣD) nonnegative, meaning that the same change of “orthant order” induced by *D* that renders X MTP_2_ also renders X positively associated. The same *D* is also such that DPD becomes nonnegative, as well as all partial correlations $d_{i}R_{ij.C}d_{j}$ for any subset $C\subset X\setminus \left\{X_{i},X_{j}\right\}$. The notion of inverse balance is referred to as *signed MTP*_2_ in Lauritzen et al. (2019). Given a p.d. covariance matrix Σ and its concentration matrix $K={\text{Σ}}^{-1}$, then checking inverse balance is a purely graphical condition, better tested on the graph of the partial correlation matrix P=2I−H: X is inverse balanced if and only if G(P) is balanced. In fact by construction, the “dominance” of the diagonal part encoded in the definition of M-matrix is already ensured by the fact that *H* is p.d., hence, only the sign of the off-diagonal entries must be checked, and having ${\left[DHD\right]}_{ij}\leq 0$ for all i≠j for some *D* amounts to having DPD nonnegative, i.e., G(P) balanced (or “non-frustrated”, as it is called in Malioutov et al. (2006)). Since

$${\text{MTP}}_{2}\;\begin{array}{l} \Rightarrow \\ ⇍ \end{array}\;\text{ positive association}$$

it is also

$$\text{ inverse balance}\;\begin{array}{l} \Rightarrow \\ ⇍ \end{array}\;\text{ balance}.$$

#### Using balance and inverse balance for checking sample data consistency

Consider a sample correlation matrix *R*. By construction, correlation matrices are symmetric $R_{ij}=R_{ji}$, while the “true” interaction graph is a DAG, i.e., if an edge $X_{i}\to X_{j}$ exists, then $X_{j}\to X_{i}$ cannot be another edge of the DAG. Since a correlation matrix is symmetric, its graph G(R) is always undirected. Any pairwise sample-based measure of association between two variables (correlation, mutual information, entropy, etc.) is by construction symmetric, hence so is the graph built on such measure. *R* symmetric reflects the fact that correlation is not causation.

The sample correlation obtained from the data is also typically full, and so is G(R). When applied to our protein-coding units, this implies that all variables pairs are connected by an undirected edge, even those that are not so in the underlying Bayesian network, like *a* and *p*, or pairs of *a*, or pairs of *s*. In particular, the correlation graph G(R) does not normally coincide with the moral graph associated to the DAG, and does not respect the Markov conditions of the original Bayesian network. It is this passage from the “true” topology of the DAG to the undirected and fully connected topology of G(R) that provides an opportunity for checking consistency of the data, as it induces the cycles whose balance helps us discriminate the reliable data from the unreliable ones.

The simplest cycle on G(R) is a triangle of nodes $X_{i}$, $X_{j}$ and $X_{k}$. On this triangle, if we associate an empirical regulatory action to each edge of G(R) by taking the corresponding value of *R*, then it is easy to realize that if G(R) is balanced the 3 regulatory actions are compatible with each other, while if G(R) is not balanced, then at least one of these empirical regulations is incompatible with the others. For instance, if in the data $X_{i}$ and $X_{j}$ are positively correlated, but $X_{j}$ and $X_{k}$ are negatively correlated, then one expects that $X_{i}$ and $X_{k}$ should also be negatively correlated (balanced case) while if instead $X_{i}$ and $X_{k}$ are positively correlated (unbalanced case) then the 3 time series of $X_{i}$, $X_{j}$ and $X_{k}$ are incompatible with each other and should not belong to the same “true” regulatory graph. This test can be extended to an arbitrary sample correlation graph, and gives us a way to label certain interaction patterns as “more plausible” (candidates to be true positives) or as “less plausible” (candidates to false positives) based on the samples. On the original DAG of our protein-coding units we cannot have cycles, but whenever we consider two or more adjacent edges on the DAG then the corresponding undirected graph G(R) has cycles, and a balance test naturally follows. For instance, if $X_{i}=a$ and $X_{j}=s$ and $X_{k}=p$ are linked by edges on a DAG, then it is possible to check if the regulatory pattern of the “true” path a→s→p, deduced by the correlations $R_{as}$ and $R_{sp}$, is compatible with the corresponding “nonphysical” branch a→p deduced by the correlation $R_{ap}$, see Figure 1C of the article for an illustration. The test is purely on the sample correlations and does not lead to any causality violation on the corresponding DAG. A similar argument can be set up for any DAG, or subset of a DAG, involving at least two adjacent edges.

Since inverse balance is computed from sample concentration matrices in an analogous way, inverse balance of a DAG can also be investigated using the same principles.

#### Sample correlation balance and verification of conditional independencies

The Markov independence conditions of a DAG can be checked for empirical validity using sample correlations and sample partial correlations. In particular, we can say that

$$X_{i}⊥\;X_{j}|C\;\Leftrightarrow \;|R_{X_{i}X_{j}.C}|<\;\theta$$

whereC denotes the conditioning set and *θ* is a significance threshold obtained by e.g. setting an hypothesis test using a Fisher z-transform (Tong, 1990)

$$Z\left(X_{i},X_{j}|C\right)=\frac{1}{2}\text{ln}\left(\frac{1+R_{X_{i}X_{j}.C}}{1-R_{X_{i}X_{j}.C}}\right)$$

and then choosing

$$\theta =\text{argmax}|R_{X_{i}X_{j}.C}|\;\text{ subject to }\;\sqrt{N-\text{card}\left(C\right)-3}\;\cdot \;|Z\left(X_{i},X_{j}|C\right)|<{\text{Φ}}^{-1}\left(1-\frac{\alpha }{2}\right)$$

where Φ is the cumulative probability distribution of the multivariate Gaussian N(0,I), *N* is the sample size, and α=0.05 is the significance level. See Kalisch and Bühlmann (2007) for an identical test. Typical values we obtain for our sample size are θ∼0.16 which is a reasonable threshold for our partial correlations.

What is shown in the article is that balance of G(R) leads to verification of conditional independencies much more often than unbalance. This is a heuristic property, and indeed exceptions are visible in the data. Also for the more strict property of inverse balance the same happens. What can be made rigorous is that for inverse balance conditioning leads to an element-wise contraction towards 0 of the “residual” partial covariance.

Even if the property “balance leads to decrease of correlation upon conditioning” cannot be turned into a rigorous statement in the balanced case (not even for covariances), it is however empirically true in a large fraction of our sample data. This combines well with the previous observation that balance is an indication of data compatibility (i.e., an intrinsic test of “true positivity” of the interactions).

#### Binary classification for balance and inverse balance under varying acceptance thresholds

A natural way to read Table 3 is to think of the “verified/non-verified” condition as the true condition, and of “balance / unbalance” (or “inverse balance / inverse unbalance”) as the prediction. This would associate “False Positives” (FP) to the combination “non-verified + balanced”, and “False-Negatives” (FN) to “verified + unbalanced”. However, if we want to turn Table 3 into a binary classificator depending on a threshold, then we have to invert the role of the two conditions, taking “balance / unbalance” (or “inverse balance / inverse unbalance”) as truth and “verified / non-verified” as prediction. In fact, the latter pair is the only one depending on a threshold, here the value *θ* associated to a Fisher z-test (verification ⇔$|R_{X_{i}X_{j}.C}|<\theta$). The only practical effect is to invert the roles of FP and FN, meaning that the total amount of errors is unchanged. In this way we can evaluate the effect of continuously varying *θ* on the accuracy of the reconstruction, for instance through Receiver-Operating Characteristic (ROC) curves or Precision-Recall curves. A ROC curve plots False Positive Rates (FPR) vs True Positive Rate (TPR), where

$$\begin{array}{l} \text{FPR}=\frac{\text{FP}}{\text{FP}+\text{TN}}\;\left(\text{TN }=\text{ True Negatives}\right)\\ \text{TPR}=\frac{\text{TP}}{\text{TP}+\text{FN}}\;\left(\text{TP }=\text{ True Positives}\right) \end{array}$$

while in a Precision-Recall curve

$$\begin{array}{l} \text{Precision}=\frac{\text{TP}}{\text{TP}+\text{FP}}\\ \text{Recall}=\text{TPR}. \end{array}$$

See Figures 6E and 6F for the results.

#### List of T_H_1 related genes

The following list of gene was compiled assembling the Gene Ontology annotation for the following categories:

T-helper 1 type immune response
T-helper 1 cell differentiation
regulation of T helper 1 cell differentiation

*IKBKB, JAK2, IFNG, ANXA1, JUN, LCK, HMGB1, VAV1, SPN, CREB1, PLCG1, NFKB1, BCL3, GATA3, PIK3R1, MAPK1, PTPN6, MAP2K2, STAT5A, ZAP70, MAP2K4, RELB, MAP2K1, ITK, NFATC3, LCP2, MAP3K1, IL18R1, STAT4, PDK1, CAMK4, IL27RA, MAP3K3, TBX21*.

#### List of T-cell related genes

The following list of gene was compiled combining the list of T_H_1 related genes given above with the Gene Ontology annotation for the following categories:

positive_regulation_Tcell_differentiation
negative_regulation_Tcell_differentiation
T_cell_activation

*TESPA1, CCDC88B, PIK3CD, BTN3A1, AP3D1, IKBKB, RAB29, ZBTB7B, SART1, NCK2, JAK2, GRAP2, SASH3, SOD1, PNP, ABL1, IFNG, IL2RA, CD4, TFRC, ANXA1, CD3D, HLA-A, HLA-DPB1, ICAM1, JUN, CD5, LCK, CD2, CTSL, CD3E, LGALS1, HMGB1, CD7, CD3G, PRKAR1A, CD28, HSPD1, SRF, HLA-E, LCP1, RAC2, VAV1, SPN, CREB1, NCK1, CTLA4, IL7R, CTPS1, LGALS3, EGR1, LAG3, PLCG1, NFKB1, TNFRSF1B, ITGAL, BCL3, RPS3, GATA3, MSN, CD27, DPP4, PIK3R1, ITPKB, MAPK1, PTPN6, CD40LG, SLC7A1, CORO1A, AKT1, PRDX2, TNFSF8, PTGER4, MYH9, MAP2K2, TGFBR2, DDOST, PSMB10, CSK, STAT5A, PIK3CA, WAS, ZAP70, TNFRSF4, MAP2K4, ZFP36L2, CD151, PIK3CG, RAB27A, AIF1, NCKAP1L, B2M, RPS6, GRB2, RAC1, ADAM8, SMAD3, EFNB1, RELB, MAP2K1, PTPN11, ZFP36L1, ITK, NFATC3, LCP2, FADD, PAK2, MAP3K1, IL18R1, TCIRG1, RUNX3, CBFB, FLOT2, FGL2, STAT4, CASP8, PDK1, RHOH, BATF, CAMK4, ZC3H12A, TNFAIP8L2, IL27RA, APBB1IP, METTL3, RASAL3, ZFPM1, ZC3H8, RSAD2, DOCK2, KAT2A, GLMN, DNAJA3, MAP3K3, NDFIP1, FANCD2, TWSG1, AZI2, VSIR, NHEJ1, CD274, CHD7, STOML2, TBX21, SIT1, RIPOR2, RIPK3, SELENOK*.

## Data Availability

• RNA-seq data: available at EMBL-EBI sequencing archive ArrayExpress (https://www.ebi.ac.uk/arrayexpress/) under accession number E-MTAB-7775 (Magnusson et al., 2020). • Mass spectrometry data: available at EMBL-EBI proteomics repository PRIDE (https://www.ebi.ac.uk/pride/) under accession number PXD013361 (Magnusson et al., 2020). • ATAC-seq data: available at ArrayExpress with accession number E-MTAB-10444 (Rundquist et al., 2022). The data and the code used in this article will be shared upon request to the corresponding author.

## Supplemental information

Document S1. Figures S1–S4 and Table S1

Data S1. Processed mass-spectrometry data, see STAR Methods
