# Supplementary material for: Multi-omics protein-coding units as massively parallel Bayesian networks: Empirical validation of causality structure
Source: iScience. 2022 Mar 11;25(4):104048. doi: 10.1016/j.isci.2022.104048 (PMC8958332; doi:10.1016/j.isci.2022.104048)
Supplement: Document S1. Figures S1–S4 and Table S1 [file mmc1.pdf]

iScience, Volume *25*

Supplemental information

Multi-omics protein-coding units as massively parallel Bayesian networks: Empirical validation of causality structure

Alberto Zenere, Olof Rundquist, Mika Gustafsson, and Claudio Altafini

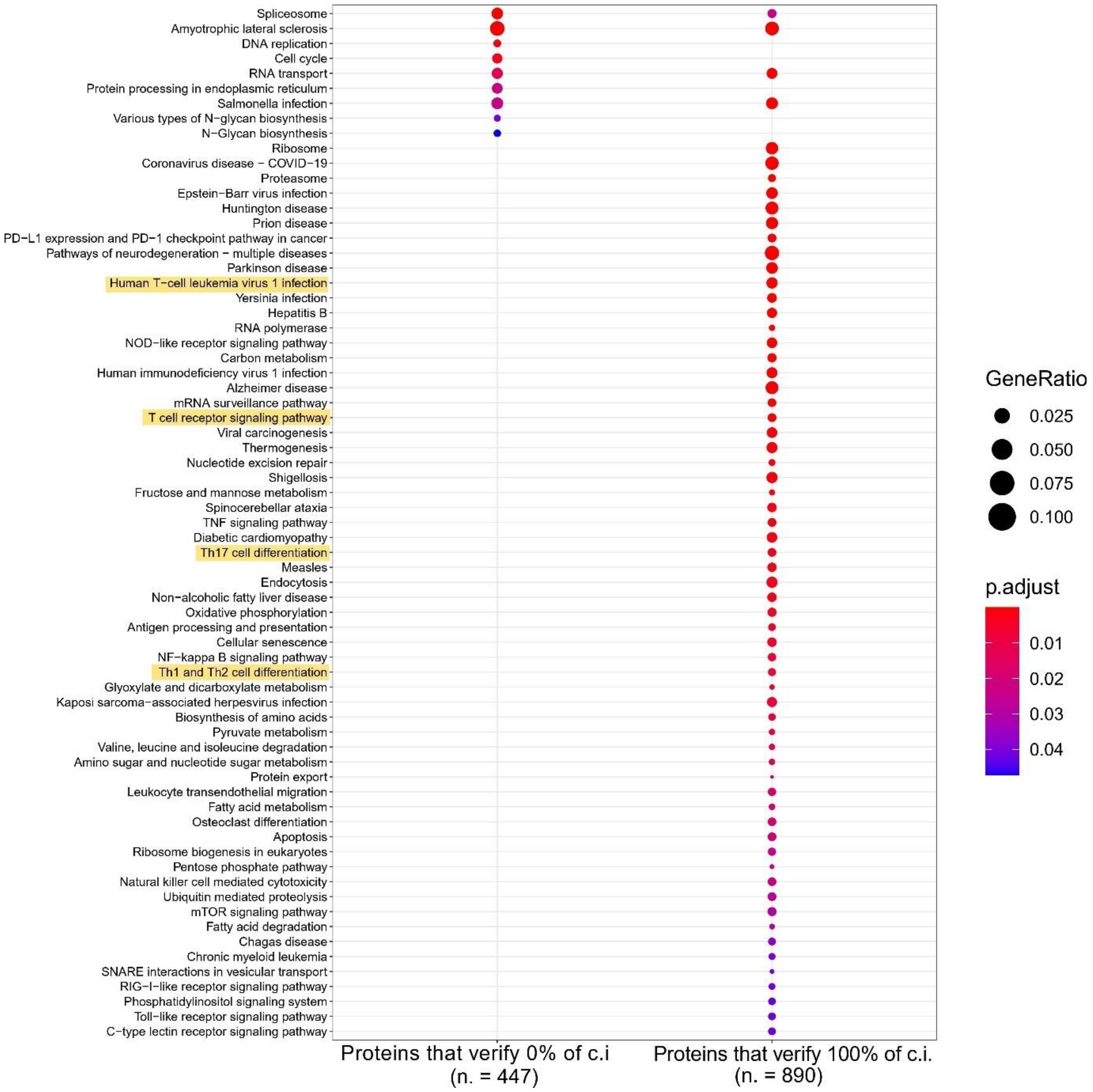

Figure S1: Ontological analysis of the protein-coding units. Related to Fig. 3(b). Left column: Proteins verifying 0% of the $a \rightarrow s$ and $a \rightarrow p$ conditional independencies; Right column: Proteins verifying 100% of the $a \rightarrow s$ and $a \rightarrow p$ conditional independencies. T-cell related pathways are highlighted in yellow.

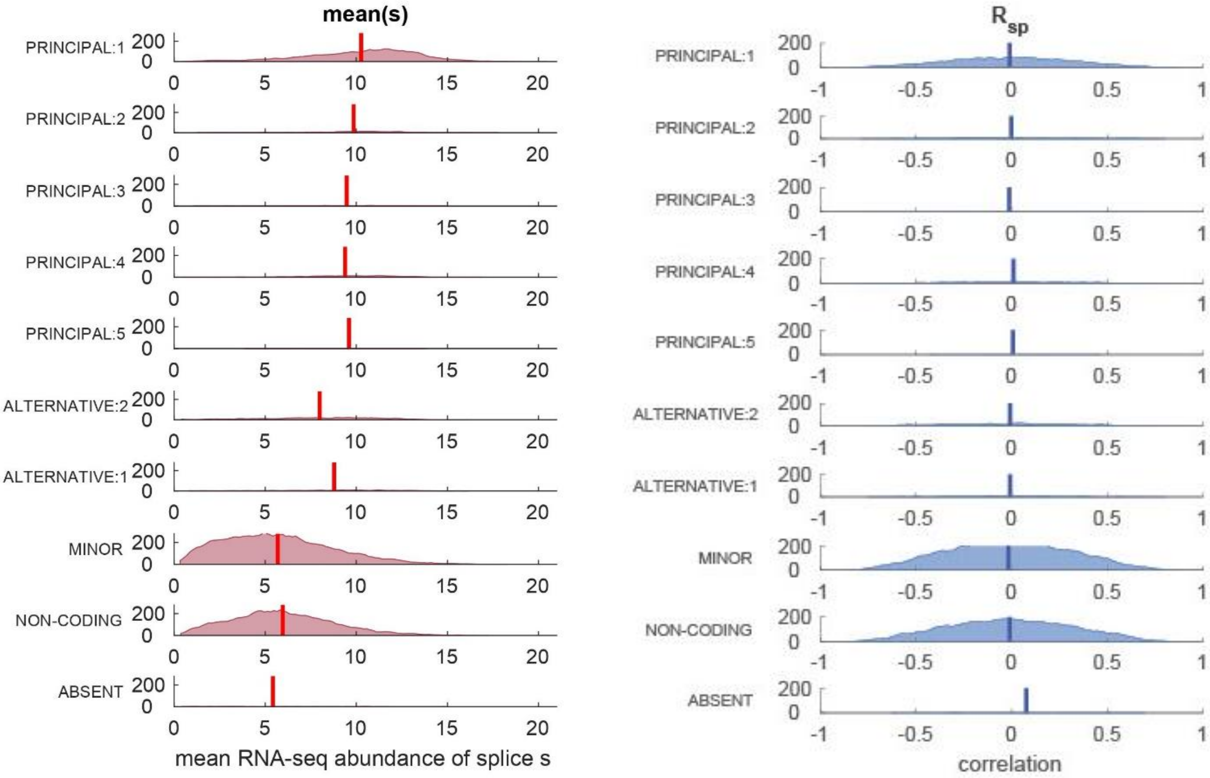

Figure S2: Mean RNA-seq expression and splice-protein correlation based on the APPRIS score. Related to Fig. 5. (A): Histogram of the mean RNA-seq expression of the splices *s* classified according to the APPRIS scores associated to *s*. Principal splices are more abundant than splices with lower classification. The red bar is the mean of the expression means. (B): Histogram of the splice - protein correlation (Rsp) classified according to the APPRIS scores associated to the splice variants *s*. All classes have Rsp with similar mean (blue bars) and variance.

Markov c.i.	total	s-s	p-p
Total	39819	38769	1050
verified	11259	11062	197
non-verified	28560	27707	853

Table S1: Number of predicted and verified conditional independencies induced by the presence of common peaks between different protein-coding units. Related to Tab. 1.

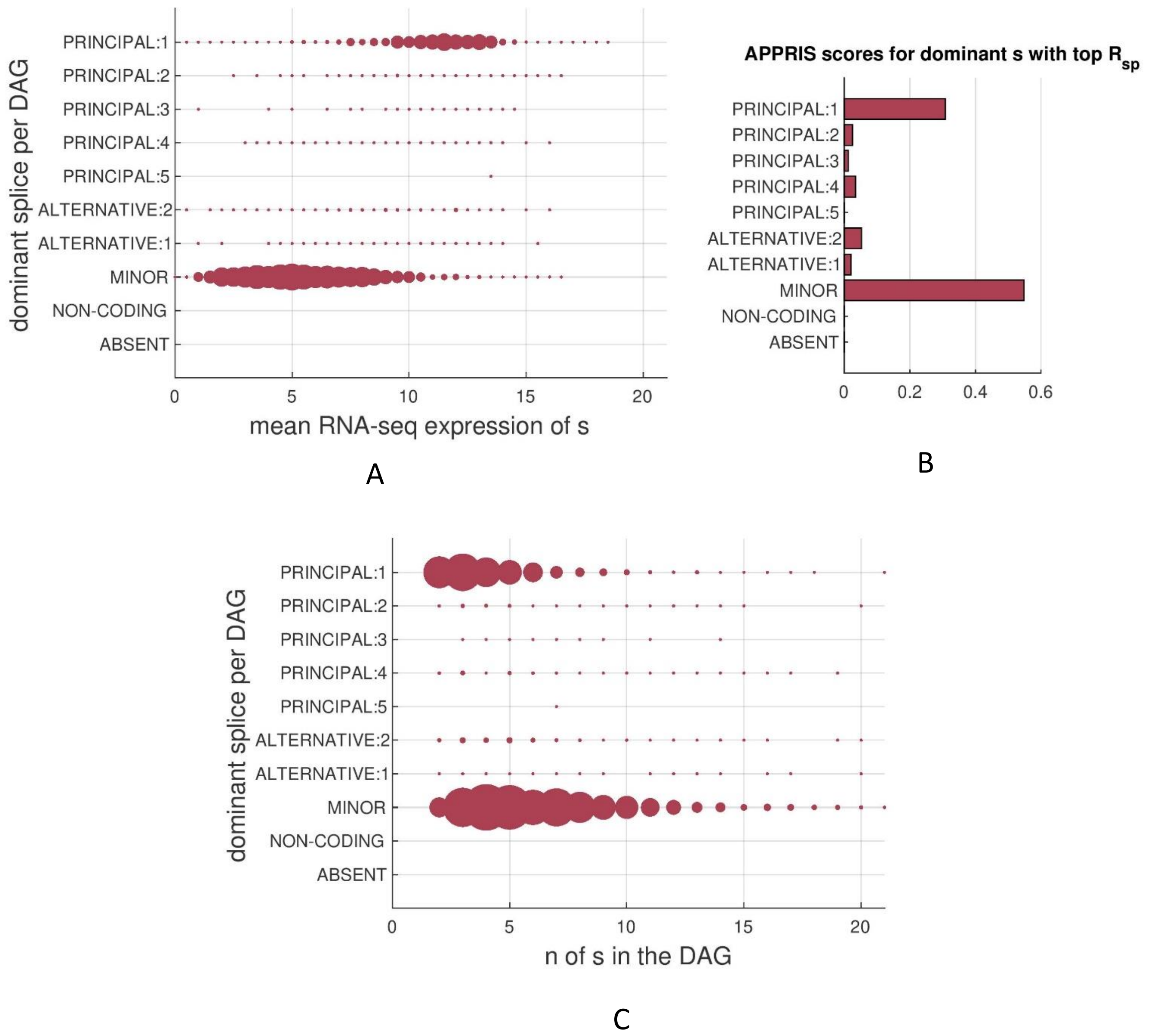

Figure S3: Analysis of the dominant splices *s* (one for each DAG). Related to Fig. 5. (A): Mean of the RNA-seq expression of the splices *s* that are dominant on each DAG, classified according to the APPRIS scores associated to *s*. (B): Distribution according to APPRIS scores of the *s* which are both dominant in a DAG and that have the highest Rsp for that DAG. (C): N. of splice variants *s* on a DAG compare to the APPRIS classification of the dominant *s* on that DAG.

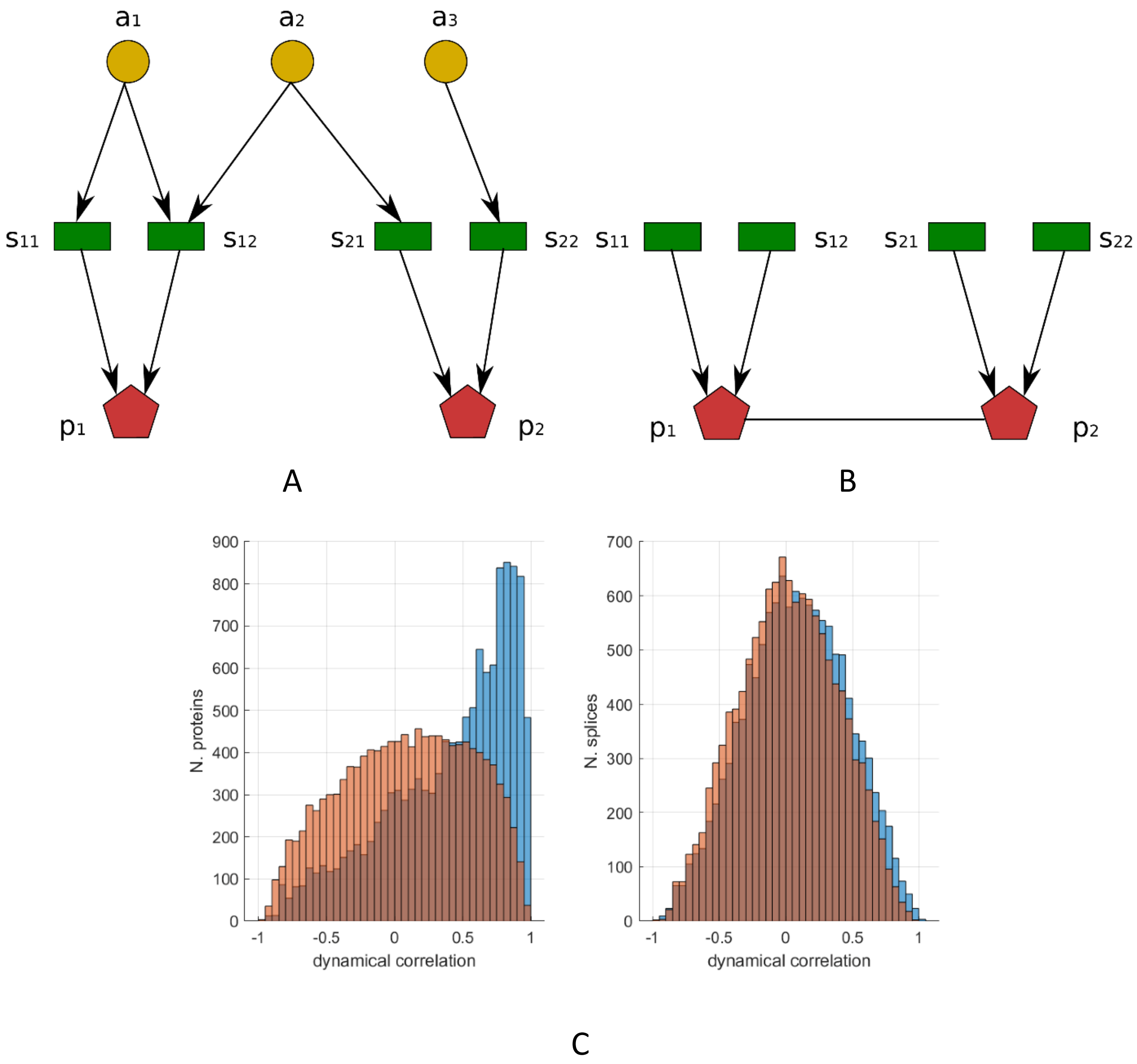

Figure S4: Overview of the analysis of coupled protein coding units. Related to Fig. 1. (A): Joint DAG of two proteins-coding units that share a common peak. a denote ATAC-seq peaks, *s* indicate splices, and p represent proteins. (B): Mixed graph obtained when two protein-coding units are connected by a *p*--*p* (undirected) edge, representing the fact that the two proteins are subunits of a protein complex. (C): Dynamical correlation between proteins or splices that are associated to the same protein complex. (Left) Dynamical correlation between proteins that belong to the same complex (blue) compared to random pairs of proteins (red). (Right) Dynamical correlation between splices associated to the same complex (blue) compared to random pairs of splices (red).
